# Ornithine enhances common bean growth and defense against white mold disease via interfering with *SsOAH* and diminishing the biosynthesis of oxalic acid in *Sclerotinia sclerotiorum*

**DOI:** 10.3389/fpls.2025.1483417

**Published:** 2025-04-04

**Authors:** Yasser Nehela, Yasser S. A. Mazrou, Nehad A. EL_Gammal, Osama Atallah, Abdelrazek S. Abdelrhim, Sumit Kumar, Temoor Ahmed, Qurban Ali, Abeer H. Makhlouf, Warda A. M. Hussain

**Affiliations:** ^1^ Department of Agricultural Botany, Faculty of Agriculture, Tanta University, Tanta, Egypt; ^2^ Business Administration Department, Community College, King Khalid University, Abha, Saudi Arabia; ^3^ Plant Pathology Research Institute, Agricultural Research Center, Giza, Egypt; ^4^ Department of Plant Pathology, Faculty of Agriculture, Zagazig University, Zagazig, Egypt; ^5^ Department of Plant Pathology, Faculty of Agriculture, Minia University, Minia, Egypt; ^6^ Institute of Agricultural Sciences, Banaras Hindu University, Varanasi, Uttar Pradesh, India; ^7^ Department of Life Sciences, Western Caspian University, Baku, Azerbaijan; ^8^ Department of Plant Biotechnology, Korea University, Seoul, Republic of Korea; ^9^ Department of Biology, College of Science, United Arab Emirates University, Al Ain, United Arab Emirates; ^10^ Department of Agricultural Botany, Faculty of Agriculture, Minufiya University, Shebeen El-Kom, Egypt

**Keywords:** ornithine, white mold, bean, antioxidant, *Sclerotinia*, OAH, oxalic acid

## Abstract

The necrotrophic fungal phytopathogen, *Sclerotinia sclerotiorum* (Lib.) de Bary, employs a multilayered strategy to infect a wide range of host plants. The current study proposed the diamine _L_-ornithine, a non-proteinogenic amino acid that promotes the synthesis of other essential amino acids, as an alternative management strategy to boost the molecular, physiological, and biochemical responses of common bean (*Phaseolus vulgaris* L.) against white mold disease caused by *S. sclerotiorum*. *In vitro* experiments showed that _L_-ornithine significantly inhibited the mycelial growth of *S. sclerotiorum* in a dose-dependent manner. Moreover, it markedly diminished the white mold severity under greenhouse conditions. Moreover, _L_-ornithine stimulated the growth of treated plants suggesting that the tested concentration of _L_-ornithine has no phytotoxicity on treated plants. Additionally, _L_-ornithine enhanced the non-enzymatic antioxidants (total soluble phenolics and flavonoids), the enzymatic antioxidants (CAT, POX, and PPO), and upregulated the gene expression of three antioxidant-associated genes (*PvCAT1*, *PvSOD*, and *PvGR*). Moreover, *in silico* analysis showed that the genome of *S. sclerotiorum* possesses a putative oxaloacetate acetylhydrolase (*SsOAH*) protein that is highly similar in its functional analysis, conserved domains, and topology with OAH from *Aspergillus fijiensis* (*AfOAH*) and *Penicillium lagena* (*PlOAH*). Interestingly, the addition of _L_-ornithine to the potato dextrose broth (PDB) medium significantly down-regulated the gene expression of *SsOAH* in the mycelium of *S. sclerotiorum*. Likewise, exogenous application of _L_-ornithine significantly down-regulated the gene expression of *SsOAH* in the fungal mycelia collected from treated plants. Finally, _L_-ornithine application significantly diminished the secretion of oxalic acid in the PDB medium as well as infected leaves. Collectively, _L_-ornithine plays a pivotal role in maintaining the redox status, in addition to boosting the defense responses of infected plants. The current study provides insights that may lead to innovative eco-friendly approaches for controlling white mold disease and mitigating its impact on common bean cultivation particularly, and other crops in general.

## 1 Introduction

White mold, caused by the necrotrophic fungus *Sclerotinia sclerotiorum* (Lib.) de Bary, is a devastating yield-limiting disease that poses a significant threat to bean (*Phaseolus vulgaris* L.) production worldwide (Bolton et al., 2006). *S. sclerotiorum* is one of the most formidable challenging soil-borne fungal phytopathogens, with a broad host range of over 600 plant species, and incites rapid host tissue maceration in a nondiscriminant manner (Liang and Rollins, 2018). Upon unfavorable conditions, it undergoes a crucial phase in its life cycle by producing black, hard, seed-like resting structures known as ‘sclerotia’ in the soil for extended periods, or it may develop in the white fuzzy growths of mycelium or the stem pith of infected plants (Schwartz et al., 2005). The ability of *S. sclerotiorum* to form sclerotia contributes to its long-term survival and the persistence of the disease in infested fields (Schwartz et al., 2005). Sclerotia contain nutrient assets and can persevere in the soil for extended periods, acting as a source of primary inoculum for subsequent infections (Schwartz et al., 2005). Under favorable conditions, sclerotia germinate to produce airborne spores that can infect all above-ground parts of the plant, including but not limited to flowers, stems, or pods (Schwartz et al., 2005).

*Sclerotinia sclerotiorum* employs a multilayered strategy to infect its host plant which involves a series of coordinated events, from sclerotia germination to symptoms development. Initially, *S. sclerotiorum* produces airborne spores called ascospores from mushroom-like structures called ‘apothecia’ that develop resting sclerotia on infected plant debris (Bolton et al., 2006). Subsequently, the fungus secretes oxalic acid, a pathogenicity factor, to manipulate the pH of the plant cell wall, facilitating enzymatic degradation and tissue invasion (Hegedus and Rimmer, 2005) and suppressing the oxidative burst of the host plant. This acidification process weakens the plant cell wall and offers a favorable environment for fungal cell wall degrading enzymes (CWDEs) to function properly and efficiently to enable the pathogen to breach the physical barrier and penetrate host tissues (Marciano et al., 1983). After penetration, *S. sclerotiorum* secretes an array of CWDEs, such as polygalacturonase and cellulases, to facilitate its spreading through the infected tissues and causing necrosis. The progression of lesions and mycelial mats contributes to the characteristic symptoms of white mold disease (Hegedus and Rimmer, 2005). Simultaneously, the host plant recognizes the pathogen-associated molecular patterns (PAMPs) through pattern recognition receptors (PRRs), triggering a series of signaling events leading to the activation of defense responses.

Despite decades of dedicated effort and due to the pathogen’s resilience, survival, and adaptability, adequate resistant germplasm is still lacking in common bean particularly, and in other economically important crops in general. Therefore, disease control is tremendously challenging and comprises integrated multifaceted strategies using a mixture of cultural practices, biological control, and chemical fungicides (O’Sullivan et al., 2021). Chemical control is the most valuable tool to manage white mold disease because fungicides can effectively control the spread of the disease, reduce the severity of infections, and minimize yield losses when applied correctly and at the right time. However, extensive use and over-reliance on fungicides can lead to the development of resistant strains of *S. sclerotiorum*, as well as negatively affect the non-target organisms, soil health, and water quality (Le Cointe et al., 2016; Ceresini et al., 2024). Therefore, the identification of eco-friendly alternatives has become a necessity.

Polyamines (PAs), such as putrescine, spermidine, spermine, and cadaverine, might be a promising alternative against soil-borne phytopathogens to reduce the use of hazardous chemical fungicides entirely or partially (Nehela et al., 2024; Yi et al., 2024). In higher plants, PAs are involved in numerous physiological processes, including, but not limited to, cell division, differentiation, and response to abiotic and biotic stresses (Killiny and Nehela, 2020). They can act as antioxidants and contribute to the scavenging of reactive oxygen species (ROS), maintaining redox homeostasis (Nehela and Killiny, 2023), induction of defense-related genes (Romero et al., 2018), modulation of multiple metabolic pathways (Nehela and Killiny, 2023), regulation of endogenous phytohormones (Nehela and Killiny, 2019), establishment of systemic acquired resistance (SAR), and modulation of the plant-pathogen interactions (Nehela and Killiny, 2020; Asija et al., 2022; Czerwoniec, 2022). It is worth mentioning that the exact mechanism(s) and the definite role of PAs in plant defense vary depending on the plant species, pathogen, and environmental conditions. The most common PAs found in plants are biosynthesized from the alkaline polyamine _L_-ornithine (Killiny and Nehela, 2020).

_L_-Ornithine plays several roles in plant growth and development. For instance, in rice (*Oryza sativa*), previous studies showed that ornithine could be associated with nitrogen reuse (Liu et al., 2018), yield formation, grain quality and aroma (Luo et al., 2020), and response to water stress (Yang et al., 2000). Moreover, exogenous application of _L_-ornithine significantly enhanced the drought tolerance in sugar beet (*Beta vulgaris*) (Hussein et al., 2019) and mitigated the salt stress in onion (*Allium Cepa*) (Çavuşoǧlu and Çavuşoǧlu, 2021) and cashew (*Anacardium occidentale*) plants (da Rocha et al., 2012). _L_-Ornithine’s potential role(s) in defending abiotic stress might be due to its involvement in proline accumulation in treated plants. For example, proline metabolism-related genes such as ornithine delta-aminotransferase (*δOAT*) and proline dehydrogenases (*ProDH1* and *ProDH2*) were reported previously to be involved in the defense against non-host strains of *Pseudomonas syringae* in *Nicotiana benthamiana* and Arabidopsis (Senthil-Kumar and Mysore, 2012). On the other hand, fungal ornithine decarboxylase (ODC) is vital for pathogen growth (Singh et al., 2020). Targeting ODC of *Fusarium oxysporum* f. sp. *lycopersici* through host-induced gene silencing (HIGS) significantly enhanced the resistance of tomato plants against fusarium wilt disease (Singh et al., 2020). However, the potential role of the exogenous application of ornithine against biotic stress such as phytopathogens is inadequately reported. More importantly, the impacts of ornithine on disease resistance and its associated biochemical and physiological events remained largely unexplored.

Understanding the intricacies of *S. sclerotiorum* infection in common bean plants is pivotal for developing effective management strategies. In the current study, we aim to unveil the potential role of the diamine, _L_-ornithine, as a key factor in enhancing the common bean’s defense mechanisms and resilience against *S. sclerotiorum*. We hypothesize that the application of _L_-ornithine plays a pivotal role in maintaining the redox status, in addition to boosting the defense responses of infected plants. We believe that the potential role of _L_-ornithine is correlated with the regulation of enzymatic and non-enzymatic antioxidant defense machinery, as well as interfering with the fungal pathogenicity/virulence factors and their associated proteins. This dual function makes _L_-ornithine a promising candidate for sustainable strategies to mitigate the impact of white mold disease and fortify the resilience of common bean plants in the face of this formidable fungal pathogen. The current study provides insights that may lead to innovative eco-friendly approaches for controlling white mold disease and mitigating its impact on bean cultivation.

## 2 Materials and methods

### 2.1 Plant material and treatments

Throughout the current study, the sensitive commercial cultivar Giza 3 of common bean (*Phaseolus vulgaris* L. cv. Giza 3) was used as an experimental plant material. Healthy seeds were generously provided by the Department of Food Legumes Research, Field Crops Research Institute (FCRI), Agricultural Research Center (ARC), Egypt. Five seeds were sown in plastic pots (35 cm inner diameter and 50 cm in depth) filled with *S. sclerotiorum*-infested soil under greenhouse conditions (25 ± 2°C, 75 ± 1% RH, and 8 h light/16 h dark photoperiod). However, 7-10 days post-sowing (dps), seedlings were thinned and only two uniform seedlings with fully expanded trifoliate leaves were left in each pot. All pots were irrigated every other week and fertilized monthly according to the recommendations for this cultivar.

To prepare a 500 mg/L stock solution of the diamine _L_-ornithine (also known as (+)-(*S*)-2,5-Diaminovaleric acid; Sigma-Aldrich, Darmstadt, Germany), 50 mg was initially dissolved in a final volume of 100 mL sterilized distilled water. Subsequently, the stock solution has been diluted and used for subsequent experiments. Briefly, six serial concentrations of _L_-ornithine (12.5, 25, 50, 75, 100, and 125 mg/L) were tested *in vitro* individually. Besides, sterilized distilled water was used as a negative control (Mock), whereas the commercial fungicide ‘Rizolex-T’ 50% WP (Tolclofos-Methyl 20% + Thiram 30%; KZ–Kafr El Zayat Pesticides and Chemicals, Kafr El-Zayat, Gharbia, Egypt) was used as a positive control. Five concentrations of the commercial fungicide ‘Rizolex-T’ were tested *in vitro* (2, 4, 6, 8, and 10 mg/L).

### 2.2 Pathogen isolation and identification

Samples of common bean stems and pods showing typical symptoms of white mold (disease severity: 10-30%) were collected from commercial farms. While most of the infected plant materials were identified by variety/cultivar (the sensitive commercial cultivar Giza 3), others, particularly those obtained from local markets, were of unknown variety. Initially, collected infected materials were surface sterilized with 0.5% NaOCl for three min, then washed several times using sterilized distilled water, and dried between sterilized filter papers to remove the extra water. Subsequently, infected organs were chopped into small pieces from the intermediate tissues (between healthy and infected ones), cultured on potato dextrose agar (PDA) medium, and incubated at 25 ± 2°C for 5 days under 12-hour light/12-hour dark cycles to encourage sclerotia formation. The hyphal tips method was also used to purify the fungal isolates from the mixed or contaminated cultures. The purified fungal isolates were first identified based on their cultural morphology characteristics, and then the identification was confirmed using microscopic features as *S. sclerotiorum*. Finally, the pathogenicity of all purified isolates was tested on the sensitive commercial cultivar Giza 3 of common bean to fulfill Koch’s postulates.

Moreover, the identification of the most aggressive isolate of *S. sclerotiorum* (isolate #3) was further confirmed based on the sequencing of the Internal transcribed spacer (ITS) region as described by (White et al., 1990; Baturo-Ciesniewska et al., 2017). Briefly, the isolate was grown on potato dextrose broth (PDB) and incubated at 25 ± 2°C for 5-7 days. Then, the fungal mycelium was harvested, filtered through cheesecloth, washed twice with sterile water, and dried using sterile filter paper. The genomic DNA was extracted using a Quick-DNA™ Fungal/Bacterial Miniprep Kit (Kuramae-Izioka, 1997; Atallah et al., 2022, 2024). Subsequently, ITS of the rDNA was amplified using a specific primer pair ITS1/ITS4 (TCCGTAGGTGAACCTGCGG TCCTCCGCTTATTGATATGC; Expected size: 540 bp) (Baturo-Ciesniewska et al., 2017). The purified PCR product was sent for sequencing (Aoke Dingsheng Biotechnology Co., Beijing, China). Sanger sequencing was used to perform the two-directional sequencing of the ITS rDNA sequence. Subsequently, a Nucleotide-Nucleotide Basic Local Alignment Search Tool (BLASTn) was used to compare the assembled query sequence with the most recent available data in GenBank and the National Center for Biotechnology Information website (NCBI, http://www.ncbi.nlm.nih.gov/gene/). Alignment was carried out using ClustalW through the Molecular Evolutionary Genetics Analysis software package (MEGA-11; version 11) (Kumar et al., 2024) to align the query sequence with other 20 *Sclerotinia* strains/isolates retrieved from the recently available data in NCBI GenBank (**Supplementary Table S1**). Evolutionary analysis was inferred using the Maximum Likelihood method and General Time Reversible model (Nei and Kumar, 2000) of nucleotide substitutions, and the tree with the highest log likelihood was shown. The initial tree for the heuristic search was selected by choosing the tree with the superior log-likelihood between a Neighbor-Joining (NJ) tree (Kumar et al., 2024) and a Maximum Parsimony (MP) tree. The NJ tree was generated using a matrix of pairwise distances computed using the General Time Reversible model (Nei and Kumar, 2000).

### 2.3 *In vitro* antifungal activity of _L_-ornithine against *S. sclerotiorum*

The antifungal activities of _L_-ornithine, as well as the ‘Rizolex-T’ fungicide, were tested *in vitro* using the agar diffusion method. Briefly, a proper volume of _L_-ornithine stock solution (500 mg/L) was finely mixed with 10 mL PDA medium to obtain the final concentration of 12.5, 25, 50, 75, 100, and 125 mg/L. Moreover, five concentrations of ‘Rizolex-T’ fungicide (2, 4, 6, 8, and 10 mg/L) and sterilized distilled water were used as controls. After media solidification, a 4 mm-diameter mycelial plug of freshly prepared culture of *S. sclerotiorum* was transferred into the middle of the petri dish, incubated at 25 ± 2°C until the mycelial growth covered the whole control plate, then the fungal growth was recorded. The percentage of radial growth inhibition of *S. sclerotiorum* was calculated using Equation 1:


(1) $$\%\text{Radial growth inhibition}=\frac{\text{Diameter of mycelial growth of control}-\text{Diameter of mycelial growth of treatment}}{\text{Diameter of mycelial growth of control}}\times 100$$

The experiment was repeated twice with six biological replicates per control/treatment and five pots (two plants per pot) for each biological replicate. Each biological replicate was analyzed twice (two technical replicates) to ensure the accuracy, reliability, and reproducibility of our experimental findings. Additionally, the half-maximal inhibitory concentration (IC_50_) and IC_99_ were calculated using probit regression analysis (Prentice, 1976).

### 2.4 Greenhouse experiments

Two successive pot experiments were carried out to evaluate the potential of _L_-ornithine under greenhouse conditions. Briefly, pots were filled with sterilized clay-sandy soil (3:1) and inoculated with a freshly prepared culture of *S. sclerotiorum*. Initially, actively growing cultures of the most aggressive isolate of *S. sclerotiorum* (isolate #3) were produced by bisecting a sclerotium, placing it face down on PDA, and incubating at 25°C for 4 days under continuous darkness (24 h) to encourage mycelial growth. Then, four 5-mm diameter agar plugs from the leading edge were used to inoculate 100 g of a sterile mixture of wheat and rice bran (1:1, v/v), then all flasks were incubated at 25 ± 2°C for 5 days under 12-hour light/12-hour dark cycles to encourage sclerotia formation. Prior to the soil inoculation, all flasks were thoroughly mixed to ensure homogeneity. Subsequently, 100 g of the colonized bran mixture was used to inoculate each pot to ensure consistent pathogen concentration. Inoculated pots were directly watered to activate fungal growth and left under greenhouse conditions for 7 days.

Later, five seeds of the cultivar Giza 3 were sown in each pot. For _L_-ornithine- and ‘Rizolex-T’ fungicide-treated pots, disinfected seeds were initially soaked in aqueous solutions of both compounds at a final concentration of the IC_99_ (about 250 mg/L, and 50 mg/L, respectively) for two hours, then air-dried for one-hour before sowing. On the other hand, seeds were soaked in sterilized distilled water as a negative control. At 10 dps and right before the first irrigation, seedlings were thinned and only two uniform seedlings were left in each pot. Furthermore, to warrant the infection with *S. sclerotiorum* stems of bean plants at the same developmental stage (10 dps) were cut in two different positions using a sterilized scalpel, and approximately 0.5 g of the colonized bran mixture was placed into each wound, then a high level of humidity was maintained to encourage infection and disease development for all inoculated plants. A similar wounding procedure was performed on the control plants and the same amount (0.5 g) of the sterile non-colonized bran mixture was placed into the wounds and maintained under high humidity conditions to simulate the environment for disease development, ensuring consistency between the treatments.

For treatments, bean seedlings were watered via soil drench with 500 mL of aqueous solutions of _L_-ornithine (250 mg/L) or ‘Rizolex-T’ fungicide (50 mg/L), then the treatment was repeated three times in total at 10 days intervals. Mock-treated control was irrigated with 500 mL of sterilized distilled water. All treatments were maintained under the greenhouse conditions (25 ± 2°C, 75 ± 1% RH, and 8 h light/16 h dark photoperiod). All pots were irrigated every other week and fertilized monthly using a balanced NPK fertilizer (20-20-20, in addition to 3.6% Sulfur and Microelements TE; Zain Seeds, Egypt) at a concentration of 3-4 g/L, applied via foliar spray according to the recommendations for this cultivar and the manufacturer’s instructions. Unless otherwise mentioned, the fully expanded, mature leaves (2^nd^ and 3^rd^ leaves from the top) were collected at 72 hours post-treatment (hpt) from each biological replicate, homogenized, mixed, and stored at −80°C for further analyses including, but not limited to, *in situ* histochemical localization of oxidative stress indicators, lipid peroxidation, enzymatic and non-enzymatic antioxidants, and gene expression.

### 2.5 Assessment of white mold disease and vegetative parameters

The severity of white mold disease was evaluated weekly till 21 days post-inoculation (dpi) using a 1-9 scale (**Supplementary Table S2**) according to Petzoldt and Dickson scale (Petzoldt and Dickson, 1996) as modified by Terán et al. (2006). Briefly, the stem and branches of the bean plant were examined starting from the inoculated site and observed for lesion progression along the internodes and nodes. Then, the distance of the lesion from the point of inoculation to its furthest point along the stem or branch was measured and a 1-9 score (based on the lesion position) was assigned, where a score (1) was assigned for no visible infection near the inoculant site, whereas scores (2-9) were assigned to progressively increasing lesion size and advancement across nodes/internodes (**Supplementary Table S2**). Subsequently, the severity of white mold disease was converted into a percentage using Equation 2:


(2) $$\text{Disease severity}(\%)=\frac{\text{Total points score}}{\text{Total number of plants}\times \text{highest score}}\times 100$$

Moreover, the area under the disease progress curve (AUDPC) was calculated according to (Shaner and Finney, 1977) which was recently adapted for the white mold disease in the common bean (Chauhan et al., 2020) using Equation 3:-


(3) $$AUDPC=\sum_{i=1}^{n}\frac{\left(Y_{i}+\;Y_{i+1}\right)}{2}\;\times (t_{i+1}\;-\;t_{i})$$

Where, *Y_i_* = Disease severity at time *t_i_*, *Y_i+1_* = Disease severity at the next time point *t_i+1_*, *t_i_* = Time of the first measurement (in days), *t_i+1_* = Time of the next measurement (in days), and *n* = Total number of time points or observations. Vegetative parameters of bean plants including plant height (cm), number of branches per plant, and number of leaves per plant were recorded weekly for 21 days for all biological replicates.

### 2.6 Photosynthetic pigment contents

Leaf samples (2^nd^ and 3^rd^ fully developed leaves from the top) were collected at 45 dps (15 days post-last treatment) from each biological replicate. Each biological replicate contains five pots (two plants per pot). Approximately 500 mg of ground tissues were used to extract the photosynthetic pigments (chlorophyll *a*, chlorophyll *b*, and carotenoids) using 80% acetone in the dark at 4°C. After 24 hours, samples were centrifuged and the supernatant was collected and the chlorophyll *a*, chlorophyll *b*, and carotenoids contents were colorimetrically assessed by measuring the absorbance at three different wavelengths (A_470_, A_646_, and A_663_ nm) using a UV-160A spectrophotometer (Shimadzu, Japan) as described by (Lichtenthaler, 1987). Finally, the content of photosynthetic pigments was calculated using the following Equations 4–6 as described by Lichtenthaler (1987).


(4) $$\text{Chlorophyll }a\;({\text{mg g}}^{-1}\text{ FW})=12.25\times {\text{A}}_{663}-2.79\times {\text{A}}_{646}\;$$

(5) $$\text{Chlorophyll }b\;({\text{mg g}}^{-1}\text{ FW})=21.50\times {\text{A}}_{646}-5.10\times {\text{A}}_{663}$$

(6) $$\text{Carotenoids }({\text{mg g}}^{-1}\text{ FW})=\frac{1000\times {\text{A}}_{470}-1.82\times \text{Chl }a-85.02\times \text{Chl }b\;}{198}$$

### 2.7 Oxidative stress indicators

#### 2.7.1 In situ histochemical localization of hydrogen peroxide (H_2_O_2_) and superoxide anion (O_2_^•−^)

Leaves (2^nd^ and 3^rd^ fully developed leaves from the top) were collected from each biological replicate at 72 hours post-treatment (hpt) for *in situ* histochemical localization of hydrogen peroxide (H_2_O_2_) and superoxide anion (O_2_^•−^). Each biological replicate contains five pots (two plants per pot). Each biological replicate was analyzed twice (two technical replicates) to ensure the accuracy, reliability, and reproducibility of the method. H_2_O_2_ and O_2_^•−^ were determined using 0.1% 3,3′-Diaminobenzidine (DAB; Sigma–Aldrich, Darmstadt, Germany) or nitro blue tetrazolium (NBT; Sigma–Aldrich, Darmstadt, Germany) as described by Romero-Puertas et al. (2004) and Adám et al. (1989), respectively, with slight modifications. For *in situ* histochemical localization of H_2_O_2_, leaflets were vacuum-infiltrated with 0.1% DAB in 10 mM tris buffer (pH 7.8), then incubated under light at room temperature for 60 min. Leaflets were bleached in 0.15% (w/vol) TCA in ethanol: chloroform 4:1 (v/v) (Al-Gomhoria Company for medicines and medical supplies, Cairo, Egypt), then illuminated until the development of brown color. Likewise, leaflets were vacuum-infiltrated with 10 mM potassium phosphate buffer (pH 7.8) containing 0.1 w/v % NBT for *in situ* histochemical localization of O_2_^•−^. Leaflets were incubated under light at room temperature for 20 min before being bleached as described above, then illuminated until the appearance of dark blue/purple spots. The intensity of the developed brown color (as an indicator of H_2_O_2_) or blue/purple color (as an indicator of O_2_^•−^) was evaluated using the Fiji version of ImageJ image processing package (http://fiji.sc; accessed on 7 March 2024).

#### 2.7.2 Assessment of lipid peroxidation

Malondialdehyde (MDA; as a lipid peroxidation marker) was assessed as described by Du and Bramlage (1992) with slight modification. Leaves (2^nd^ and 3^rd^ fully developed leaves from the top) were collected from each biological replicate at 72 hours post-treatment (hpt). Each biological replicate contains five pots (two plants per pot). Each biological replicate was analyzed twice (two technical replicates) to ensure the accuracy, reliability, and reproducibility of the method. Briefly, 0.5 g of ground leaf tissue was used to extract MDA using 20% trichloroacetic acid (TCA; MilliporeSigma, Burlington, MA, USA) containing 0.01% butyl hydroxyl toluene (BHT; Sigma–Aldrich, Saint Louis, MO, USA). Subsequently, MDA was colorimetrically determined in the supernatants by measuring the absorbance at 532 and 600 nm using a UV-160A spectrophotometer (Shimadzu, Japan), then expressed as nmol g^−1^ FW.

### 2.8 Non-enzymatic antioxidants

For the assessment of non-enzymatic and enzymatic antioxidants, leaves (2^nd^ and 3^rd^ fully developed leaves from the top) were collected from each biological replicate at 72 hours post-treatment (hpt). Each biological replicate contains five pots (two plants per pot). Each biological replicate was analyzed twice (two technical replicates). Both leaves were ground together using liquid nitrogen and subjected directly for the analysis of enzymatic, and non-enzymatic antioxidants, total amino acids, proline content, gene expression, and the quantification of oxalic acid.

#### 2.8.1 Total soluble phenolics

Total soluble phenolics were determined using the Folin-Ciocalteu reagent (Sigma–Aldrich, Saint Louis, MO, USA) as previously described by Kähkönen et al. (1999) with minor modifications. Briefly, about 0.1 g of homogenized leaf tissues were extracted using 20 mL of 80% methanol for 24 h in the dark, centrifuged, and the supernatant was collected. For the reaction mixture, 0.1 mL of the sample extract was mixed with 0.5 mL of the Folin-Ciocalteu reagent (10%), vortexed for 30 seconds, and let stand for 5 minutes in the dark. Subsequently, 0.5 mL of 20% sodium carbonate (Na_2_CO_3_; Al-Gomhoria Company for medicines and medical supplies, Cairo, Egypt) solution was added to each tube, mixed thoroughly, then incubated in the dark for one hour at room temperature. After incubation, the absorbance of the reaction mixture was measured at 765 nm using a UV-160A spectrophotometer (Shimadzu, Japan). A calibration curve of gallic acid (Fisher Scientific, Hampton, NH, USA) was used to determine the concentration of total soluble phenolics in the sample extracts, then expressed as mg of gallic acid equivalents per gram of fresh weight (mg GAE g^−1^ FW).

#### 2.8.2 Total soluble flavonoids

Total soluble flavonoids were determined using Djeridane’s method (Djeridane et al., 2006) with slight modifications. Briefly, 0.3 ml of methanolic extract as described above was mixed with 0.3 of 5% aluminum chloride (AlCl_3_; Fisher Scientific, Hampton, NH, USA) solution, vigorously mixed, then incubated for 5 min at room temperature, then 0.3 mL of 10% potassium acetate (Al - Gomhoria Company for medicines and medical supplies, Cairo, Egypt) solution was added, mixed thoroughly, and incubated at room temperature for 30 minutes in the dark. After incubation, the absorbance of the reaction mixture was measured at 430 nm using a UV-160A spectrophotometer (Shimadzu, Japan). A calibration curve of rutin (TCI America, Portland, OR, USA) was used to determine the concentration of total soluble flavonoids in the sample extracts, then expressed as mg of rutin equivalents per gram of fresh weight (mg RE g^−1^ FW).

### 2.9 Total amino acids and proline content

The total free amino acids in bean leaves were determined using a modified ninhydrin (Thermo Scientific Chemicals, Waltham, MA, USA) reagent, as described by Yokoyama and Hiramatsu (2003) and improved by (Sun et al., 2006). Briefly, 0.1 g of ground tissues were extracted with a pH 5.4 buffer, and then 200 μL of the supernatant was reacted with 200 μL of ninhydrin (2%) and 200 μL of pyridine (10%; Spectrum Chemical, New Brunswick, NJ, USA), incubated for 30 min in a boiling-water water bath, then cooled, and measured at 580 nm using a UV-160A spectrophotometer (Shimadzu, Japan). On the other hand, proline was determined using Bates’s method (Bates et al., 1973). Proline was extracted with 3% sulfosalicylic acid (Thermo Scientific Chemicals, Waltham, MA, USA), centrifuged, and then 0.5 mL of the supernatant was mixed with one mL of glacial acetic acid (Fisher Scientific, Hampton, NH, USA) and ninhydrin reagent, then incubated at 90°C for 45 min, then cooled, and measured at 520 nm using the same spectrophotometer described above. Calibration curves of glycine and proline (Sigma–Aldrich, Saint Louis, MO, USA) standards were used to determine the total free amino acids and proline content, respectively, in the leaves extract and expressed as mg g^−1^ FW.

### 2.10 Antioxidant enzymes activity

To determine the enzymatic activity of antioxidant-associated enzymes, about 500 mg of homogenized tissues were extracted using 3 mL of 50 mM Tris buffer (pH 7.8) containing 1 mM EDTA-Na_2_ (Sigma–Aldrich, Saint Louis, MO, USA) and 7.5% polyvinylpyrrolidone (PVP; Sigma–Aldrich, Saint Louis, MO, USA), centrifuged at 10000× *g* for 20 min under cooling (4°C), and the supernatant (crude enzyme extract) was collected (El-Nagar et al., 2023; Osman et al., 2023). Subsequently, the enzymatic activity of catalase (CAT) was assessed according to the method of Aebi (1984) with slight modifications (El-Nagar et al., 2023; Osman et al., 2023) after the reaction with 2 mL of 0.1 M sodium phosphate buffer (pH 6.5; Sigma–Aldrich, Saint Louis, MO, USA) and 100 μL of 269 mM H_2_O_2_ solution. The enzymatic activity of guaiacol-dependent peroxidases (POX) was determined using the method of Harrach et al. (2008) with slight modifications (El-Nagar et al., 2023; Osman et al., 2023) after the reaction with 2.2 mL of 100 mM sodium phosphate buffer (pH 6.0), 100 µL guaiacol (TCI chemicals, Portland, OR, USA), and 100 µL of 12 mM H_2_O_2_. Whereas the enzymatic activity of polyphenol oxidase (PPO) was determined according to the method of Malik and Singh (1980) with slight modifications (El-Nagar et al., 2023; Osman et al., 2023). After the reaction with 3 mL catechol (Thermo Scientific Chemicals, Waltham, MA, USA) solution (0.01 M), freshly prepared in 0.1 M phosphate buffer (pH 6.0). CAT activity was measured by following the decomposition of H_2_O_2_ at 240 nm (A_240_), POX activity was measured by following the increase in the absorption at 436 nm (A_436_), whereas PPO activity was measured by recording the fluctuations in the absorbance at 495 nm (A_495_) every 30 seconds for 3 min using a UV-160A spectrophotometer (Shimadzu, Japan).

### 2.11 Gene expression of antioxidant-associated genes of common bean

The transcript levels of three antioxidant-associated genes, including peroxisomal catalase (*PvCAT1*; GenBank accession number: KF033307.1), superoxide dismutase (*PvSOD*; GenBank accession number: XM_068639556.1), and glutathione reductase (*PvGR*; GenBank accession number: KY195009.1) were assessed from common bean leaves (2^nd^ and 3^rd^ fully developed leaves from the top) at 72 hpt after last treatment using real-time RT-qPCR. Briefly, RNA was extracted using a Simply P Total RNA Extraction Kit (catalog number BSC52S1; BioFlux, Bioer technology, China), according to the manufacturer’s procedure. Then, cDNA was synthesized using a TOP script™ cDNA Synthesis Kit as described in the manufacturer’s protocol. The primer sequences for the three genes mentioned above are listed in **Supplementary Table S3**. *PvActin-3* (GenBank accession number: XM_068616709.1) was used as a housekeeping gene, and the 2^−ΔΔCT^ method was used for the calculation of relative gene expression (Livak and Schmittgen, 2001). The stability of *Actin* was confirmed previously under biotic (incompatible interaction between common bean and fungus *Colletotrichum lindemuthianum*) as well as abiotic (drought; salinity; cold temperature) stresses (Borges et al., 2012).

### 2.12 *In silico* analysis of oxaloacetate acetylhydrolase (*SsOAH*) protein from *S. sclerotiorum*

Initially, genome-wide *in silico* analysis of oxaloacetate acetylhydrolase (OAH) protein in *S. sclerotiorum* was carried out using the protein-protein BLAST (BLASTp 2.15.0+) tool (Altschul et al., 1997, 2005). Briefly, OAH from *Aspergillus fijiensis* CBS 313.89 (*AfOAH*; taxid:1191702; GenBank Accession No. XP_040799428.1; 342 aa) and *Penicillium lagena* (*PlOAH*; taxid:94218; GenBank Accession No. XP_056833920.1; 316 aa) were used as query sequences to match their homologous proteins from *S. sclerotiorum* (taxid:5180). BLASTp was carried out based on recently available data about the *S. sclerotiorum* genome on GenBank, National Center for Biotechnology Information website (NCBI, http://www.ncbi.nlm.nih.gov/gene/).

Moreover, evolutionary analysis and phylogenetic tree of predicted OAH genes from *S. sclerotiorum* (*SsOAH*), as well as *AfOAH* from *A. fijiensis* CBS 313.89 and *PlOAH* from *P. lagena* were inferred by using the Maximum Likelihood method and JTT matrix-based model (Jones et al., 1992) in MEGA11 (Tamura et al., 2021). The phylogenetic tree was combined with multiple protein sequence alignment analysis of all predicted OAH genes from *S. sclerotiorum* (*SsOAH*), as well as the query sequences using Constraint-Based Alignment Tool (COBALT; for multiple protein sequences (https://www.ncbi.nlm.nih.gov/tools/cobalt/re_cobalt.cgi) (Papadopoulos and Agarwala, 2007). Furthermore, ClustalW (http://www.genome.jp/tools-bin/clustalw) was used to align the top-matched AA sequences of *SsOAH* from *S. sclerotiorum* with the query sequences (*AfOAH* and *PlOAH*) (Larkin et al., 2007) and ESPript tool (version 3.0; https://espript.ibcp.fr/ESPript/ESPript/index.php) was used to visualize conserved regions in the alignment.

Additionally, functionally representative domains and conserved sites of the predicted *SsOAH* from *S. sclerotiorum* were interactively classified into families using the InterPro tool (https://www.ebi.ac.uk/interpro/) (Blum et al., 2021). Finally, three-dimensional (3D) structure modeling of predicted *SsOAH* from *S. sclerotiorum* was carried out using the Protein Homology/analogy Recognition Engine (Phyre2-server version 2.0; http://www.sbg.bio.ic.ac.uk/~phyre2/html/page.cgi?id=index) (Kelley et al., 2015), then confirmed using the SWISS-MODEL server (https://swissmodel.expasy.org/) (Biasini et al., 2014). The predicted 3D structures (PDB format) were interactively visualized using the UCSF-Chimera package (version 1.15; https://www.cgl.ucsf.edu/chimera/) (Pettersen et al., 2004).

### 2.13 Gene expression of oxaloacetate acetylhydrolase (*SsOAH*) from *S. sclerotiorum*

The transcript levels of oxaloacetate acetylhydrolase (*SsOAH*; GenBank accession number: XM_001590428.1) were assessed from *S. sclerotiorum* mycelia using real-time RT-qPCR. Briefly, *S. sclerotiorum* was inoculated into PDB-containing flasks and incubated on an incubator shaker (Model I2400, New Brunswick Scientific Co., Edison, NJ, USA) at 150 rpm for 24 hours at 25 ± 2°C under continuous darkness (24 h) to encourage mycelial growth, then treated with _L_-ornithine- and ‘Rizolex-T’ fungicide at a final concentration of the IC_50_ (about 40, and 3.2 mg/L, respectively), then the incubation was continued at the same conditions for another 24 hours. After incubation, cultures were centrifuged at 2500 rpm for 5 min and the supernatant (fungal mycelia) was collected for gene expression analysis. Likewise, fungal mycelia were collected at 0, 24, 48, 72, 96, and 120 hpt from infected plants that produced a white mold that forms cottony mycelium on the surface of infected tissue. RNA was extracted from the fungal mycelium, and then cDNA was synthesized as described above. The primer sequences for *SsOAH* are listed in **Supplementary Table S3**. *SsActin* (GenBank accession number: XM_001589919.1) was used as a housekeeping gene, and the 2^−ΔΔCT^ method was used for the calculation of relative gene expression (Livak and Schmittgen, 2001).

### 2.14 Quantification of oxalic acid

Oxalic acid was determined in both potato dextrose broth (PDB) containing the fungal pathogen, *S. sclerotiorum*, as well as the plant samples according to the method of Xu and Zhang (2000) with slight modifications. Briefly, *S. sclerotiorum* isolates were inoculated into PDB-containing flasks and then incubated on an incubator shaker (Model I2400, New Brunswick Scientific Co., Edison, NJ, USA) at 150 rpm for 3-5 days at 25 ± 2°C under continuous darkness (24 h) to encourage mycelial growth. After incubation, the fungal cultures were initially filtered through Whatman filter paper No. 1, then centrifuged at 2500 rpm for 5 min to remove any mycelial debris. The supernatant was collected and stored at 4°C for further quantification of oxalic acid. Approximately 0.1 g of ground tissues were extracted three times (2 ml each) using distilled water to prepare the plant sample. Subsequently, samples were centrifuged at 2500 rpm for 5 min and the supernatant was filtered dryly through Whatman No. 1 paper, then collected for further analysis.

For oxalic acid quantification, the reaction mix was prepared in a test tube fitted with a glass stopper in the order of 0.2 mL of sample (or PDB culture filtrate or standard oxalic acid solution), 0.11 ml of bromophenol blue (BPB, 1 mM; Fisher Chemical, Pittsburgh, PA, USA), 0.198 ml of 1 M sulfuric acid (H_2_SO_4_; Al - Gomhoria Company for medicines and medical supplies, Cairo, Egypt), and 0.176 ml of 100 mM potassium dichromate (K_2_Cr_2_O_7_; TCI chemicals, Portland, OR, USA), then the solution was made up to the mark using 4.8 ml of distilled water, vigorously mixed, and placed in the water bath immediately at 60°C. After 10 minutes, the reaction was quenched by adding 0.5 ml sodium hydroxide solution (NaOH; 0.75 M). The absorbance of the reaction mix was measured at 600 nm (A_600_) using a UV-160 spectrophotometer (Shimadzu, Japan). PDB and distilled water were used as a blank control for quantification in culture filtrate or plant samples, respectively. A calibration curve of oxalic acid (Thermo Scientific Chemicals, Waltham, MA, USA) was used to determine the concentration of oxalic acid in the culture filtrate and expressed as *μ*g oxalic acid per mL PDB medium (*μ*g.mL^−1^) as well as in the leaves extract which was expressed as *μ*g oxalic acid per gram fresh weight (*μ*g.g^−1^ FW).

### 2.15 Statistical analyses

Throughout the study, unless otherwise stated, all experiments were arranged in a completely randomized design (CRD) with six biological replicates per treatment and five pots (two plants per pot) for each biological replicate. Biological replicates were analyzed twice (two technical replicates). Technical replicates were used to test the reproducibility within the same experiment, but they were not used in the statistical analysis to avoid the possibility of pseudoreplication. Data were statistically analyzed using the analysis of variance (ANOVA), followed by the Tukey-Kramer honestly significant difference (HSD) test (*p* ≤ 0.05). For the *in vitro* experiments, probit analysis was used to calculate the IC_50_ and IC_99_ with 95% confidence intervals.

## 3 Results

### 3.1 Characterization of *S. sclerotiorum* isolates from common bean plants

**Figure 1:**
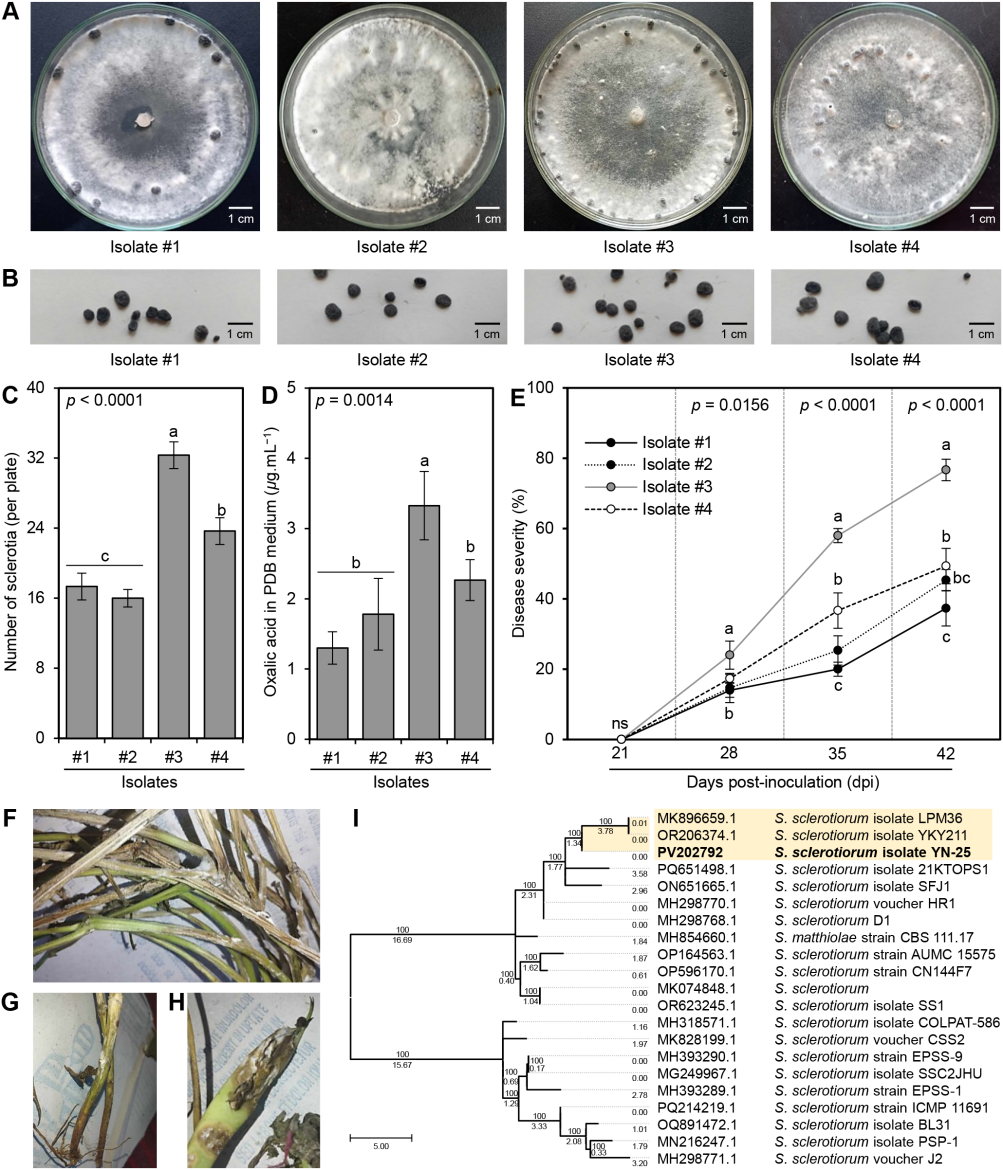
Characterization and Pathogenicity of *S. sclerotiorum* isolates from common bean plants. **(A)** Mycelial growth of four *S. sclerotiorum* isolates on PDA media at sclerotia formation stage, **(B)** Sclerotia of four *S. sclerotiorum* isolates, **(C)** Number of sclerotia (per plate), **(D)** Oxalic acid secretion in PDB media (*μ*g.mL^−1^), and **(E)** Disease severity (%) of four *S. sclerotiorum* isolates on the sensitive commercial cultivar Giza 3 of common bean under greenhouse conditions. Values represent the means ± standard deviation (means ± SD) of five biological replicates (*n* = 5). Different letters indicate statistically significant differences between treatments (*p* < 0.05). (**F–H**) Typical white mold symptoms on above-ground parts stems, and pods, respectively, after 10 days post-inoculation (dpi) with isolate #3. **(I)** Evolutionary analysis by the Maximum Likelihood method of the internal transcribed spacer (ITS) region of *S. sclerotiorum* isolate #3 in comparison to 20 reference isolates/strains retrieved from The National Center for Biotechnology Information (NCBI) database (https://www.ncbi.nlm.nih.gov/). Numbers above the cluster lines indicate the site coverage (%), whereas, numbers below the cluster lines indicate the branch length.

A total of 4 isolates were collected from different bean fields in El-Gharbia Governorate, Egypt. On the PDA medium, all isolates developed creamy white mycelia that rapidly became cottony white (**Figure 1A**), then turned beige or brownish at the stage of sclerotia formation. Generally, the sclerotia were hard-bodied, black-colored, globose to irregular in shape, and measured 5.2 to 7.7 mm in length and 3.4 to 5.3 mm in diameter (**Figure 1B**). Although the four isolates showed a peripheral sclerotia pattern that developed at the edge of the culture media 10-12 days post incubation at 25 ± 2°C (**Figure 1A**), significant differences were observed in the number of sclerotia per plate between them (*P* < 0.001) with superiority for isolate #3 (32.33 ± 1.53 sclerotia per plate; **Figure 1C**). Likewise, isolate #3 produced more oxalic acid in PDB than other isolates (3.33 ± 0.49 *μ*g.mL^−1^; **Figure 1D**). Isolate #3 showed typical morphological and microscopic characteristics with the phytopathogenic fungus *S. sclerotiorum*. For instance, on PDA, colonies of isolate #3 were fast growing with milky white (**Figure 1A**), and reverse beige or little salmon-buff color and took 6-7 days to fully cover the surface of a 9-cm Petri dish when incubated at 25 ± 2°C. Collectively, isolate #3 was identified as *S. sclerotiorum* based on the above-mentioned morphological and microscopic features.

Moreover, to confirm pathogenicity, Koch’s postulates were fulfilled using the four obtained isolates of *S. sclerotiorum* to inoculate the sensitive commercial cultivar Giza 3 of common bean under greenhouse conditions (**Figure 1E**). Although all obtained fungal isolates were pathogenic and were able to infect common bean (cv. Giza 3) producing typical white mold symptoms on all above-ground parts (**Figure 1F**), particularly stems (**Figure 1G**) and pods (**Figure 1H**) after 10 days post inoculation (dpi), isolate #3 was the most aggressive isolate in two separate experiments. Isolate #3 had the highest disease severity (%) on bean plants (24.0 ± 4.0, 58.0 ± 2.0, and 76.7 ± 3.1 at 7, 14, and 21 dpi, respectively; **Figure 1F**).

The identification of the most aggressive *S. sclerotiorum* isolate #3 was further confirmed based on the sequencing of the Internal transcribed spacer (ITS) region (**Figure 1I**). The phylogenetic analysis between isolate #3 and 20 reference isolates/strains showed high similarity (over 99%) between them. It is worth mentioning that *S. sclerotiorum* – isolate #3 (533 bp) showed high similarity with the American isolate *S. sclerotiorum* isolate LPM36 (GenBank accession No. MK896659.1; 540 bp) isolated from the dry pea seed and the Chinese isolate *S. sclerotiorum* isolate YKY211 (GenBank accession No. OR206374.1; 548 bp) the causal agent of stem rot of stock (*Matthiola incana*) which all were clustered separately at the top of the dendogram (**Figure 1I**). The new sequence was deposited in the NCBI database and named ‘*S. sclerotiorum* - isolate YN-25’ (GenBank Accession No. PV202792). Accordingly, it was noticeable that isolate #3 was the most aggressive isolate; consequently, it was chosen for all subsequent experiments.

### 3.2 *In vitro* antifungal activity of _L_-ornithine

**Figure 2:**
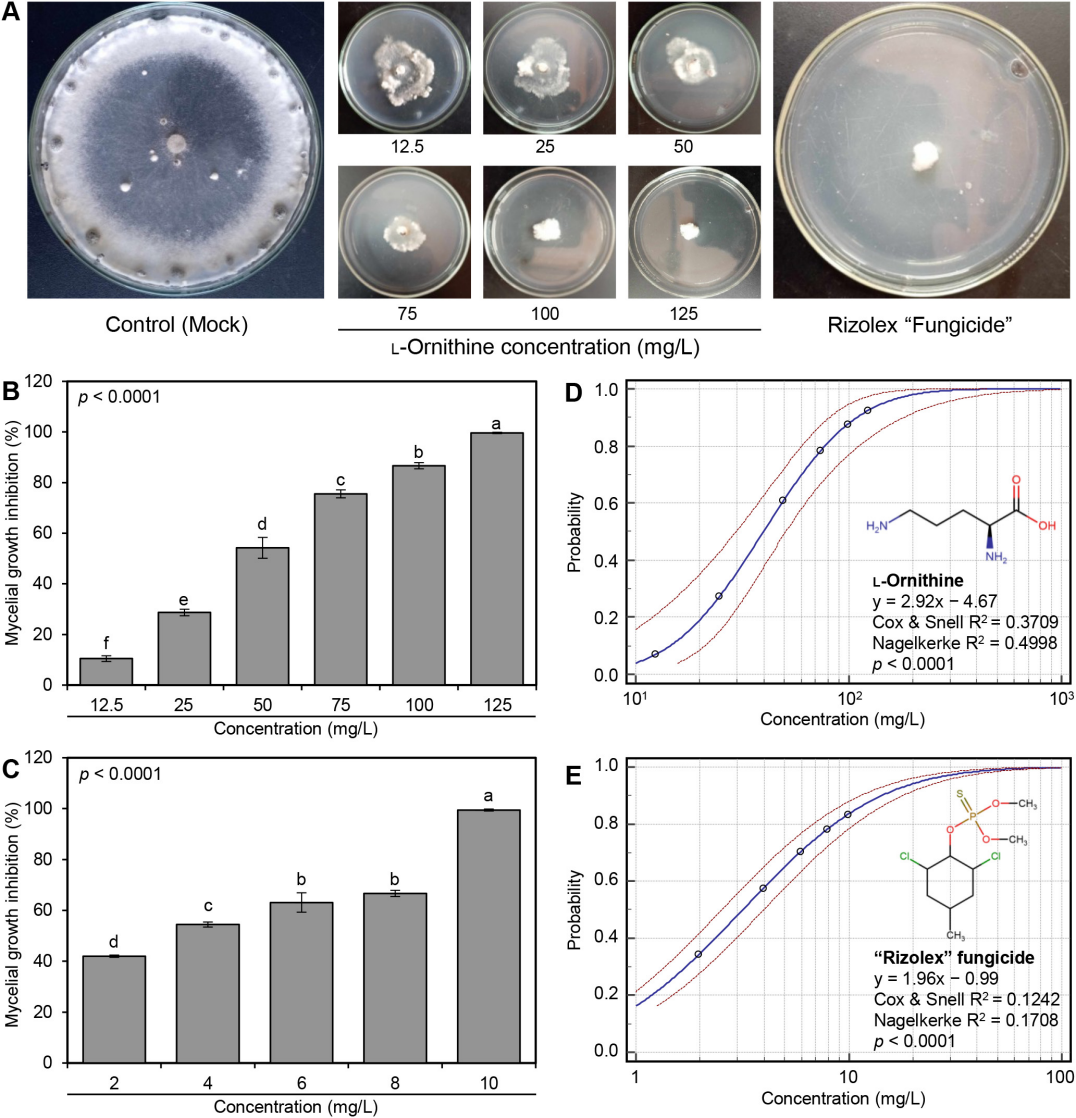
*In vitro* antifungal activity of _L_-ornithine against *S. sclerotiorum*. **(A)** Antifungal activity of serial concentrations of _L_-ornithine against *S. sclerotiorum* in comparison with the commercial fungicide ‘Rizolex-T’ (10 mg/L). **(B, C)** Mycelial growth inhibition (%) of *S. sclerotiorum* after the treatment with different concentrations of _L_-ornithine (12.5, 25, 50, 75, 100, and 125 mg/L) or ‘Rizolex-T’ fungicide (2, 4, 6, 8, and 10 mg/L), respectively. Values represent the means ± standard deviation (means ± SD) of five biological replicates (*n* = 5). Different letters indicate statistically significant differences between treatments (*p* < 0.05). **(D, E)** Probit regression analysis of _L_-ornithine and the commercial fungicide ‘Rizolex-T’, respectively. The probit regression lines are presented as blue solid lines, whereas the confidence intervals (95%) are edged with dotted red lines.

The antifungal activity of serial concentrations of (12.5, 25, 50, 75, 100, and 125 mg/L) of the diamine, _L_-ornithine (Sigma-Aldrich, Darmstadt, Germany), was examined *in vitro* against *S. sclerotiorum* – isolate #3. It is worth mentioning that _L_-ornithine showed fungistatic action and progressively inhibited the mycelial radial growth of *S. sclerotiorum* in a dose-dependent manner (**Figures 2A, B**). At the highest tested concentration (125 mg/L), _L_-ornithine had the highest mycelial growth inhibition (99.62 ± 0.27%; **Figure 2B**) which was equivalent to the highest tested concentration (10 mg/L) of the commercial fungicide ‘Rizolex-T’ (99.45 ± 0.39% inhibition; **Figure 2C**), suggesting similar potency.

Moreover, the probit regression analysis was carried out and its associated plots are presented in **Table 1** and **Figures 2D, E**. Briefly, the acceptable slope value of _L_-ornithine (y = 2.92x − 4.67) and its associated significant statistics (Cox & Snell R^2^ = 0.3709, Nagelkerke R^2^ = 0.4998, and *p* < 0.0001; **Figures 2D**) compared with the commercial fungicide ‘Rizolex-T’ (y = 1.96x − 0.99, Cox & Snell R^2^ = 0.1242, Nagelkerke R^2^ = 0.1708, and *p* < 0.0001) indicates a potentiating effect on antifungal activity against *S. sclerotiorum* (**Table 1**).

**Table 1:** The half-maximal inhibitory concentration (IC_50_) and IC_99_ values (mg/L) of _L_-ornithine and the commercial fungicide ‘Rizolex-T’ against *S. sclerotiorum*.

Compound	IC_50_ (mg/L)	95% Confidence Interval		IC_99_ (mg/L)	95% Confidence Interval		Overall Model Fit		
		Lower	Upper		Lower	Upper	χ^2^	p-Value	Cox & Snell R^2^
_L_-Ornithine	39.90	29.68	50.77	249.93	157.23	609.16	278.06	< 0.0001	0.3709
‘Rizolex-T’ fungicide	3.18	2.54	3.98	48.76	38.68	61.48	66.281	< 0.0001	0.1242

### 3.3 _L_-Ornithine reduced the development of white mold on common bean plants under greenhouse conditions

**Figure 3:**
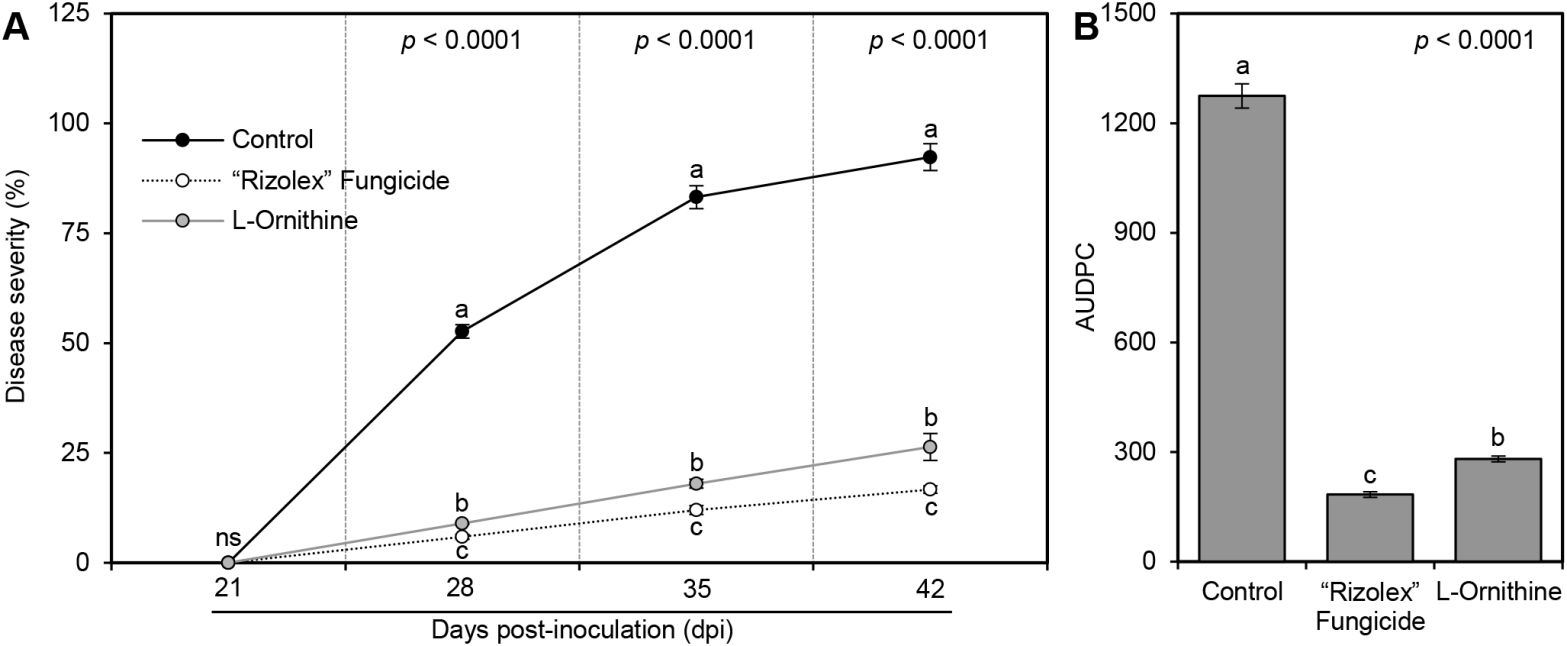
Effect of the exogenous application of _L_-ornithine on the development of white mold disease of common beans caused by *S. sclerotiorum* under greenhouse conditions. **(A)** Disease progress curves of white mold disease of common beans after the treatment with 250 mg/L _L_-ornithine. **(B)** The area under the disease progress curve (AUDPC) of white mold disease of common beans after the treatment with _L_-ornithine. Values represent the means ± standard deviation (means ± SD) of five biological replicates (*n* = 5). Different letters indicate statistically significant differences between treatments (*p* < 0.05).

Generally, _L_-ornithine (250 mg/L) significantly reduced the development and severity of white mold disease on treated common bean plants compared with non-treated *S. sclerotiorum*-infected plants (control; **Figure 3A**). Briefly, although the disease severity was progressively increased in the non-treated infected control plants (52.67 ± 1.53, 83.21 ± 2.61, and 92.33 ± 3.06%), _L_-ornithine markedly lowered disease severity (%) throughout the experiment (8.97 ± 0.15, 18.00 ± 1.00, and 26.36 ± 3.07) at 7, 14, and, 21 days post-treatment (dpt), respectively (**Figure 3A**). Likewise, the area under the disease progress curve (AUDPC) declined from 1274.33 ± 33.13 (in non-treated control) to 281.03 ± 7.95 when *S. sclerotiorum*-infected bean plants were treated with the 250 mg/L of _L_-ornithine, which was just behind the positive control 50 mg/L ‘Rizolex-T’ fungicide (183.61 ± 7.71; **Figure 3B**). The same trend was observed in the second trial.

### 3.4 _L_-Ornithine stimulated the growth of *S. sclerotiorum*-infected bean plants

**Figure 4:**
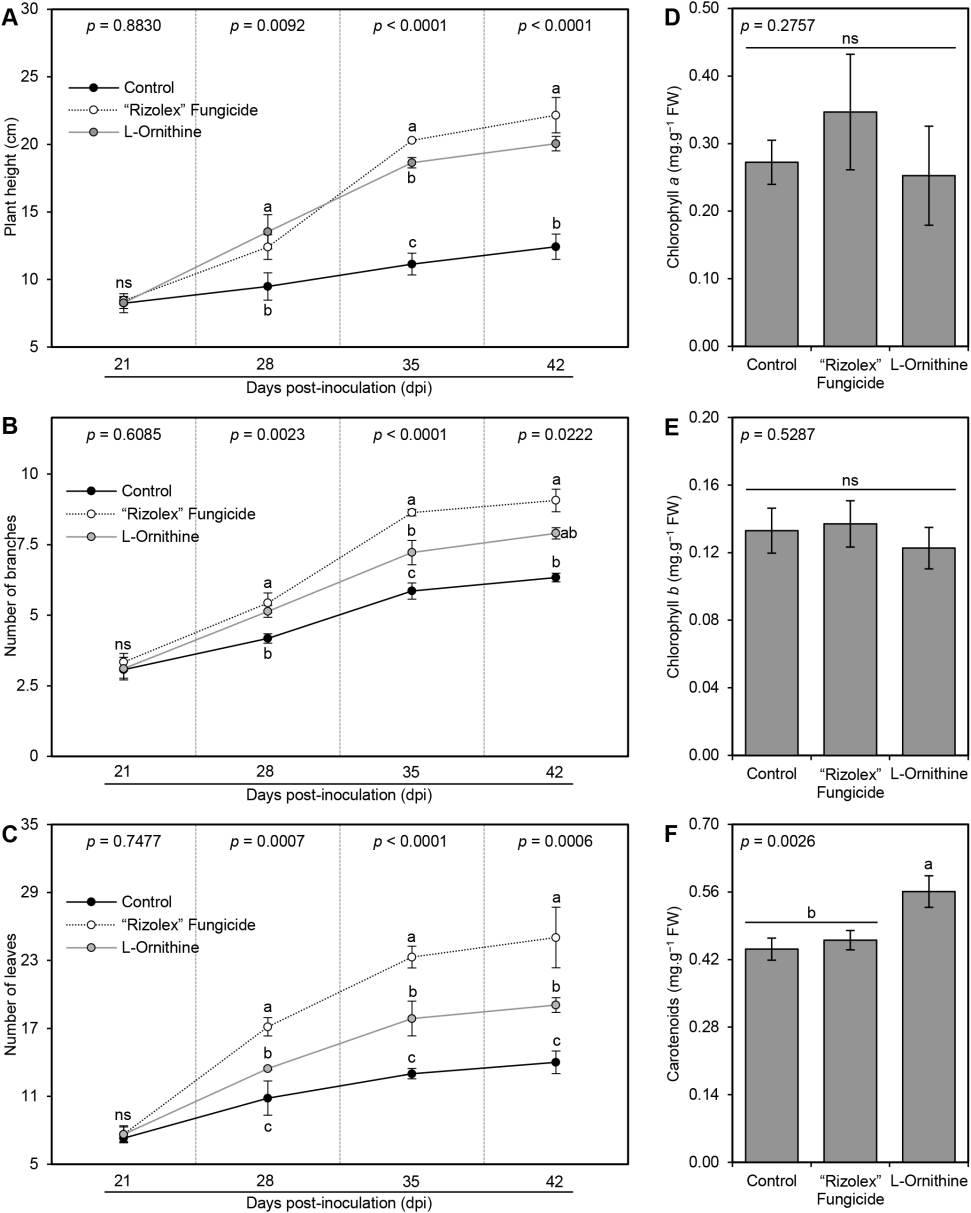
Effect of the exogenous application of _L_-ornithine on the growth features and photosynthetic pigments in the leaves of *S. sclerotiorum*-infected common beans under greenhouse conditions. **(A)** Plant height (cm), **(B)** number of branches per plant, **(C)** number of leaves per plant, **(D)** Chlorophyll *a* content (mg.g^−1^ FW), **(E)** chlorophyll *b* content (mg.g^−1^ FW), and **(F)** total carotenoids content (mg g^−1^ FW). Values represent the means ± standard deviation (means ± SD) of five biological replicates (*n* = 5). Different letters indicate statistically significant differences between treatments (p < 0.05).

Exogenous application of 250 mg/L _L_-ornithine progressively enhanced the plant height (**Figure 4A**), number of branches per plant (**Figure 4B**), and number of leaves per plant (**Figure 4C**) throughout 42 dpi. Although the commercial fungicide ‘Rizolex-T’ (50 mg/L) had the highest effect on all studied vegetative parameters, the exogenous application of _L_-ornithine (250 mg/L) came second highest when compared with non-treated control plants (**Figures 4A–C**). On the other hand, the application of _L_-ornithine had no significant effect on the content of both photosynthetic pigments chlorophyll *a* (**Figure 4D**) and chlorophyll *b* (**Figure 4E**), however, it slightly augmented the total carotenoids (0.56 ± 0.03 mg.g^−1^ FW) compared with both negative (0.44 ± 0.02 mg.g^−1^ FW) and positive controls (0.46 ± 0.02 mg.g^−1^ FW; **Figure 4F**). Collectively, these findings suggest that _L_-ornithine has no phytotoxicity on the treated bean plants, but it even stimulates their growth.

### 3.5 The diamine _L_-ornithine mitigates the oxidative stress of *S. sclerotiorum*-infected bean plants

**Figure 5:**
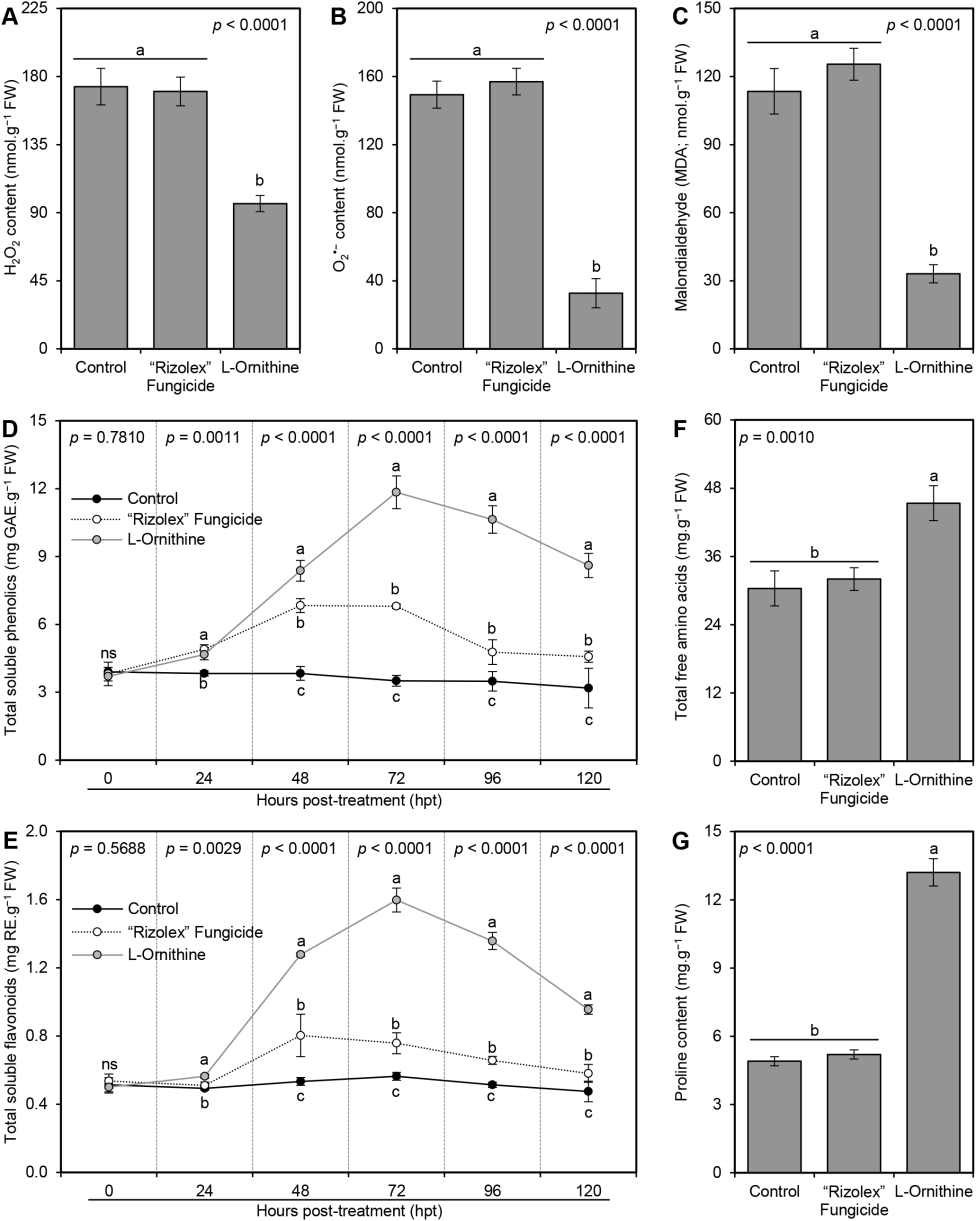
Effect of the exogenous application of _L_-ornithine on the major oxidative stress markers and non-enzymatic antioxidant defense machinery in S. sclerotiorum-infected common bean leaves at 72 hpt under greenhouse conditions. **(A)** hydrogen peroxide (H_2_O_2_; nmol g^−1^ FW) at 72 hpt, **(B)** superoxide anion (O_2_^•−^; nmol g^−1^ FW) at 72 hpt, **(C)** Malondialdehyde (MDA; nmol g^−1^ FW) at 72 hpt, **(D)** total soluble phenolics (mg GAE g^−1^ FW), **(E)** total soluble flavonoids (mg RE g^−1^ FW), **(F)** Total free amino acids (mg.g^−1^ FW) at 72 hpt, and **(G)** Proline content (mg.g^−1^ FW) at 72 hpt. Values represent the means ± standard deviation (means ± SD) of five biological replicates (*n* = 5). Different letters indicate statistically significant differences between treatments (*p* < 0.05).

*In situ* histochemical localization of the reactive oxygen species (ROS; expressed as hydrogen peroxide [H_2_O_2_]) and free radicals (expressed as superoxide anion [O_2_^•−^]) showed that exogenous application of _L_-ornithine (250 mg/L) significantly reduced the accumulation of both H_2_O_2_ (96.05 ± 5.33 nmol.g^−1^ FW; **Figure 5A**) and O_2_^•−^ (32.69 ± 8.56 nmol.g^−1^ FW; **Figure 5B**), compared to non-treated infected plants (173.31 ± 12.06 and 149.35 ± 7.94 nmol.g^−1^ FW, respectively) and those treated with 50 mg/L of the commercial fungicide ‘Rizolex-T’ (170.12 ± 9.50 and 157.00 ± 7.81 nmol.g^−1^ FW, respectively) which both accumulate higher contents of H_2_O_2_ and O_2_^•−^ at 72 hpt (**Figures 5A, B**). Similarly, TCA -based assay of malondialdehyde (MDA) suggested that *S. sclerotiorum*-infected bean plants accumulate higher MDA levels (113.48 ± 10.02 nmol.g^−1^ FW) in their leaves (**Figure 5C**). Nevertheless, exogenous application of _L_-ornithine markedly diminished lipid peroxidation as expressed by lower MDA content within treated plants (33.08 ± 4.00 nmol.g^−1^ FW).

### 3.6 _L_-Ornithine boosts the non-enzymatic antioxidant defense machinery of *S. sclerotiorum*-infected bean plants

To better understand the physicochemical mechanisms of _L_-ornithine within *S. sclerotiorum*-infected bean plants, non-enzymatic antioxidant defense machinery as expressed by total soluble phenolics (TSP; **Figure 5D**) and total soluble flavonoids (TSF; **Figure 5E**) were further investigated. Briefly, the application of _L_-ornithine (250 mg/L) induced the accumulation of TSP within treated *S. sclerotiorum*-infected bean plants to reach its highest peak at 72 hpt (11.84 ± 0.73 mg GAE.g^−1^ FW), compared to non-treated (3.51 ± 0.24 mg GAE.g^−1^ FW) and fungicide-treated controls (6.80 ± 0.10 mg GAE.g^−1^ FW) at the same time point (**Figure 5D**). Similarly, exogenous application of 250 mg/L _L_-ornithine dramatically increased the accumulation of TSF within treated *S. sclerotiorum*-infected bean plants to reach its highest peak at 72 hpt (1.60 ± 0.07 mg RE.g^−1^ FW), compared to non-treated (0.56 ± 0.02 mg RE.g^−1^ FW) and fungicide-treated controls (0.76 ± 0.06 mg RE.g^−1^ FW) at the same time point (**Figure 5E**). However, both TSP and TSF dropped thereafter when measured at 96 and 120 hpt, but still higher than both controls.

### 3.7 _L_-Ornithine enhanced the total free amino acids and proline content of *S. sclerotiorum*-infected bean plants

Generally, the application of _L_-ornithine (250 mg/L) significantly boosted the total free amino acids (45.37 ± 3.07 mg.g^−1^ FW; **Figure 5F**) and proline content (13.20 ± 0.60 mg.g^−1^ FW; **Figure 5G**) of *S. sclerotiorum*-infected bean plants compared with the non-treated control (30.38 ± 3.08 and 4.90 ± 0.20 mg.g^−1^ FW; respectively) and 50 mg/L ‘Rizolex-T’ fungicide-treated plants (32.02 ± 2.00 and 5.20 ± 0.20 mg.g^−1^ FW; respectively). Nevertheless, there were no significant differences between non-treated and fungicide-treated common bean plants in terms of total free amino acids and proline content (**Figures 5F and G**).

### 3.8 _L_-Ornithine enhances the enzymatic antioxidant defense machinery of *S. sclerotiorum*-infected bean plants

**Figure 6:**
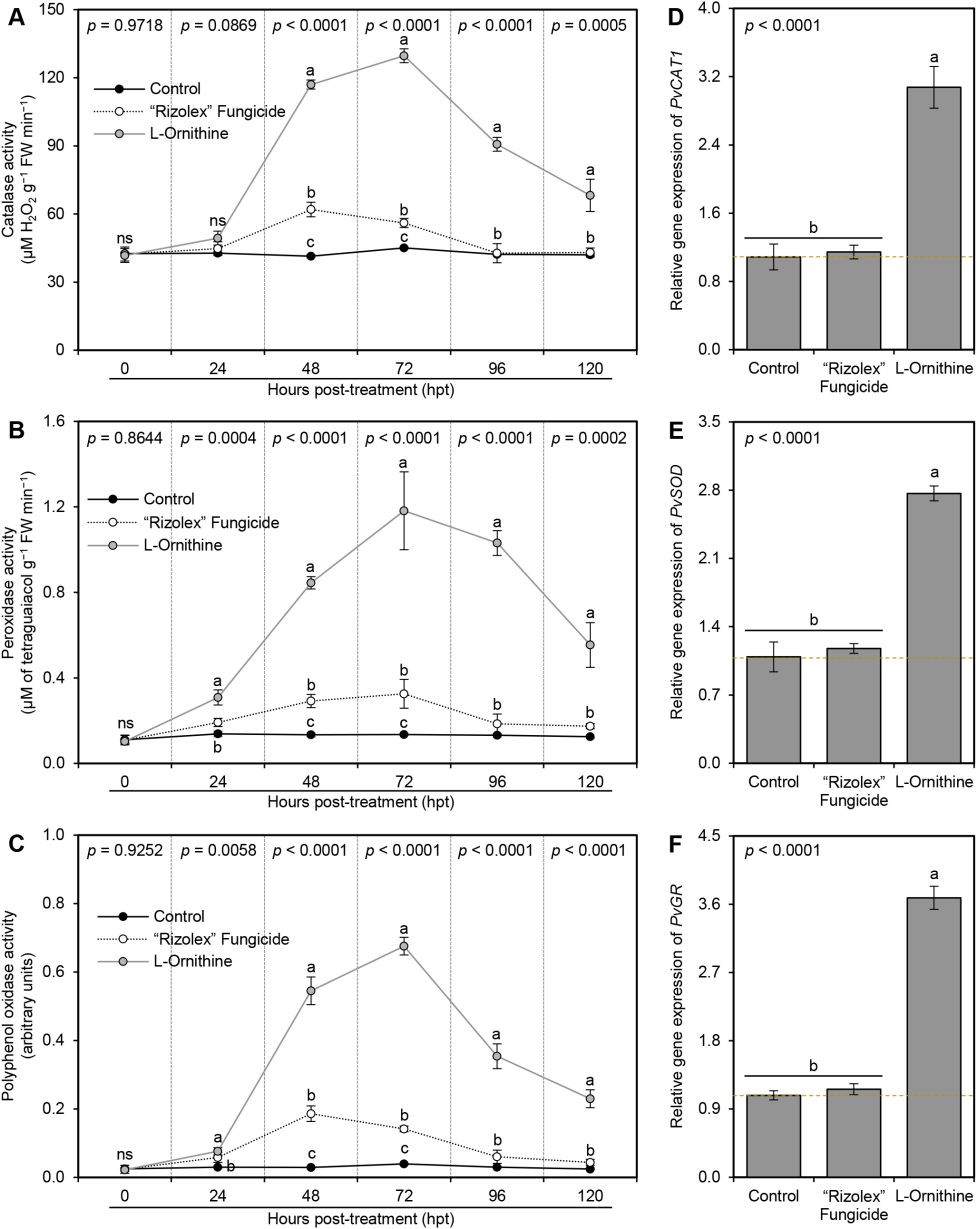
Effect of the exogenous application of _L_-ornithine on the major components of enzymatic antioxidant defense machinery of *S. sclerotiorum*-infected common beans at 72 hpt under greenhouse conditions. **(A)** catalase activity (μM H_2_O_2_ g^−1^ FW min^−1^), **(B)** peroxidase activity (μM Tetraguaiacol g^−1^ FW min^−1^), and **(C)** polyphenol oxidase activity (Arbitrary units), (D, E, and F) Relative gene expression of peroxisomal catalase (*PvCAT1*), superoxide dismutase (*PvSOD*), and glutathione reductase (*PvGR*), respectively, at 72 hpt. Values represent the means ± standard deviation (means ± SD) of five biological replicates (*n* = 5). Different letters indicate statistically significant differences between treatments (*p* < 0.05).

In agreement with its effect on non-enzymatic antioxidant defense machinery, _L_-ornithine (250 mg/L) gradually improved the enzymatic activity of catalase (CAT; **Figure 6A**), peroxidase (POX; **Figure 6B**), and polyphenol oxidase (PPO; **Figure 6C**) till they reached their highest peak at 72 hpt (129.69 ± 3.05 μM H_2_O_2_ g^−1^ FW min^−1^, 1.182 ± 0.182 μM of tetraguaiacol g^−1^ FW min^−1^, 0.676 ± 0.025 arbitrary units, respectively) compared to both controls at the same time point, then they dropped slowly at 96 and 120 hpt. Moreover, exogenous application of 250 mg/L _L_-ornithine unregulated three antioxidant-associated genes, including peroxisomal catalase (*PvCAT1*; **Figure 6D**), superoxide dismutase (*PvSOD*; **Figure 6E**), and glutathione reductase (*PvGR*; **Figure 6F**) compared to both controls.

### 3.9 The genome of *Sclerotinia sclerotiorum* possesses a putative oxaloacetate acetylhydrolase (SsOAH) protein

*In silico* analysis showed that the *S. sclerotiorum* genome possesses about six putative oxaloacetate acetylhydrolase (OAH) proteins that were similar and produced significant alignment statistics with OAH protein from *Aspergillus fijiensis* CBS 313.89 and *Penicillium lagena* (**Supplementary Table S4**). Only three of them had an identity percentage of more than 50%, including Hypothetical protein SS1G_08218 (GenBank accession no. XP_001590478.1, 338 aa, identity = 70.28 and 61.97% with OAH from *A. fijiensis* and *P. lagena*, respectively), OAH-1 (GenBank accession no. AEY84228.1, identity = 71.08 and 69.79% with OAH from *A. fijiensis* and *P. lagena*, respectively), and OAH-2 (GenBank accession no. AEY84231.1, identity = 69.02 and 65.93% with OAH from *A. fijiensis* and *P. lagena*, respectively) (**Supplementary Table S4**). It is worth mentioning that the AA sequence of both OAH-1 (249 aa) and OAH-2 (185 aa) from *S. sclerotiorum* was a little bit shorter than the query AA sequences of OAH from *Aspergillus fijiensis* (342 aa) and from *Penicillium lagena* (316 aa), but the length of AA sequences Hypothetical protein SS1G_08218 (henceforth SsOAH; 338 aa) was approximately like them. Therefore, we focused only on the SsOAH protein for further *in silico* analysis.

**Figure 7:**
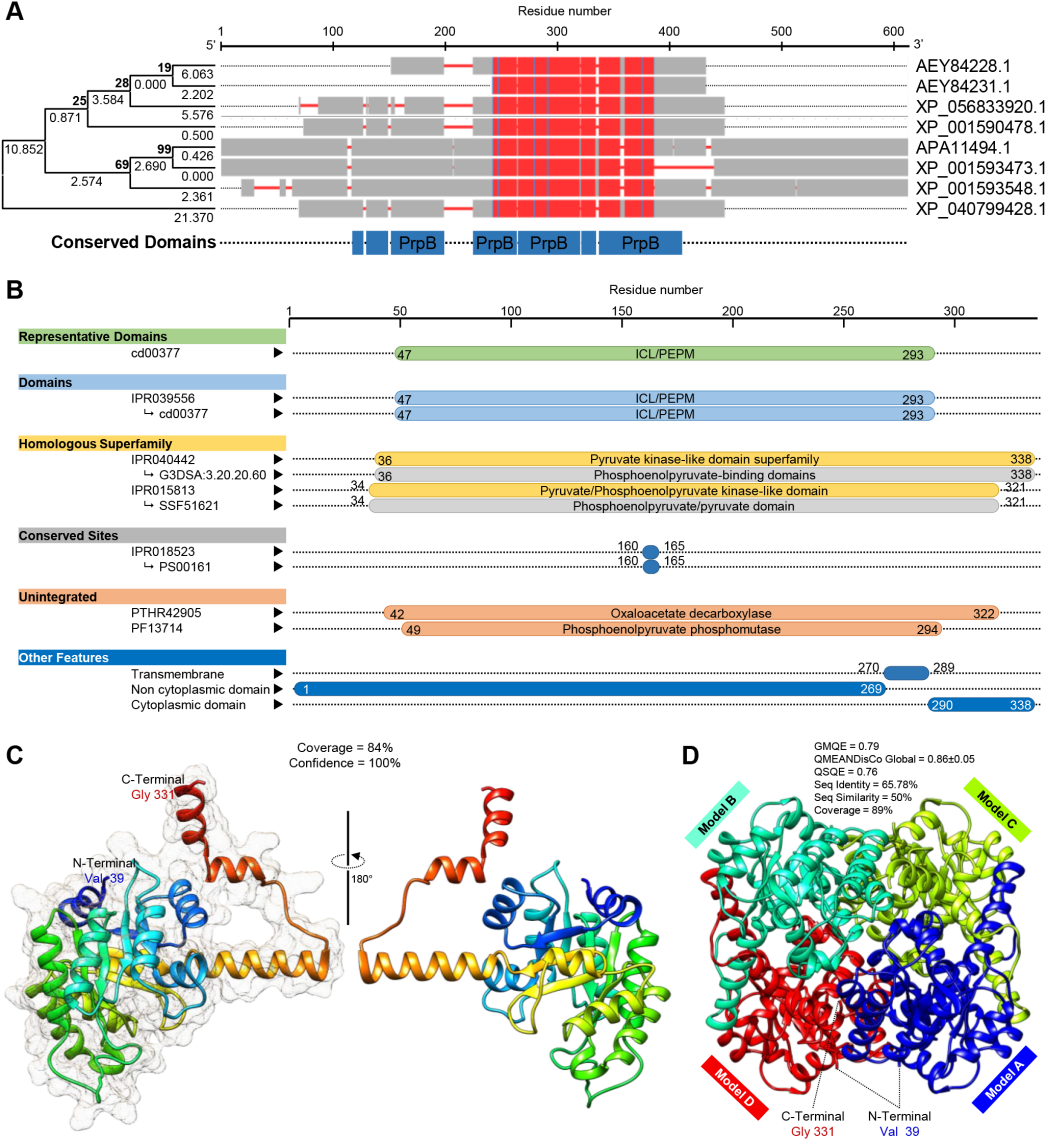
*In silico* characterization and structural modeling of oxaloacetate acetylhydrolase (*SsOAH*) protein from *S. sclerotiorum*. **(A)** Evolutionary analysis using the maximum likelihood method as conducted in Molecular Evolutionary Genetics Analysis software package (MEGA; version 11) (Kumar et al., 2024) and its associated multiple protein sequences alignments using Constraint-Based Alignment tool (COBALT) analysis. In the COBALT analysis, residues were colored using a column-based method according to their relative entropy threshold. The red color indicates highly conserved columns. The full list of genes, names, and accession numbers is available in **Supplementary Table S4**. **(B)** Functional and conserved domains analysis of *SsOAH* using the InterPro Scan tool. **(C)** The crystallographic tertiary structure and its associated mesh surface of putative *SsOAH* monomer as predicted by Phyre2 tool. The tertiary structures of *SsOAH* were predicted using the crystal structure of oxaloacetate acetylhydrolase in the protein data bank (PDB ID: 3lye.1.A) and refined using the X-ray method to 1.30 Å resolution with acceptable statistics and 100.0% confidence. Protein chains are colored according to the rainbow color spectrum, from blue (N-terminus) to red (C-terminus). **(D)** The crystallographic tertiary structure of putative *SsOAH* homo-tetramer as predicted by the SWISS-MODEL server. Both tertiary structures were visualized with the UCSF-Chimera package.

Moreover, the multiple protein sequences alignment using COBALT analysis showed that the obtained six protein sequences were relatively highly homologous to each other, as well as OAH from *A. fijiensis* and *P. lagena*, and all had a conserved 2-methylisocitrate lyase (PrpB) domain (**Figure 7A**). However, the phylogenetic analysis showed that only SsOAH was phylogenetically closer to OAH from *Penicillium lagena* while OAH from *Aspergillus fijiensis* was clustered separately at the bottom of the dendrogram (**Figure 7A**). Likewise, multiple sequence alignment showed that the SsOAH protein from *S. sclerotiorum* had high similarity and conserved sequence with both query sequences (OAH from *A. fijiensis* and *P. lagena*) (**Supplementary Figure S1**).

Additionally, the InterPro Scan tool was used for the functional analysis of proteins by classifying them into families and predicting domains and important sites of SsOAH (**Figure 7B**). Briefly, SsOAH protein has a phosphoenolpyruvate mutase (PEPM) isocitrate lyase (ICL) representative domain (ICL/PEPM, CDD entry: cd00377), as well as an ICL/PEPM domain (InterPro entry: IPR039556). Furthermore, SsOAH has a pyruvate kinase-like domain superfamily (InterPro entry: IPR040442) with phosphoenolpyruvate-binding domains (CATH-Gene3D entry: G3DSA:3.20.20.60), as well as a pyruvate/phosphoenolpyruvate kinase-like domain superfamily (InterPro entry: IPR015813) with phosphoenolpyruvate/pyruvate domain (SUPERFAMILY entry: SSF51621). It also has an isocitrate lyase/phosphorylmutase conserved site (InterPro entry: IPR018523) with an isocitrate lyase signature (PROSITE patterns entry: PS00161) **Figure 7B**). It is worth mentioning that SsOAH had an unintegrated site of isocitrate lyase/PEP mutase superfamily, oxaloacetate decarboxylase (PANTHER entry: PTHR42905) with phosphoenolpyruvate phosphomutase site (Pfam entry: PF13714). Other features of SsOAH include a region of a membrane-bound protein predicted to be embedded in the membrane (as a transmembrane site), a region of a membrane-bound protein predicted to be outside the membrane, in the extracellular region (as a non-cytoplasmic domain), and a region of a membrane-bound protein predicted to be outside the membrane, in the cytoplasm (as a cytoplasmic domain) (**Figure 7B**). Collectively, these findings suggest that the predicted SsOAH protein is highly similar in its functional analysis, conserved domains, and topology with known OAH proteins from other species.

The crystallographic three-dimensional (3D) structure of SsOAH (**Figures 7C, D**) was predicted using the crystal structure of oxaloacetate acetylhydrolase in the protein data bank (PDB ID: 3lye.1.A) and refined using the X-ray method to 1.30 Å resolution with acceptable statistics (**Figures 7C, D**; **Supplementary Figure S2**). Briefly, the Phyre2 tool showed that approximately 283 residues (84%; residues Val 39 to Gly 331) of SsOAH have been modeled with template protein with 100% confidence (**Figure 7C**). According to the Phyre2-based analysis, the predicted 3D structure of SsOAH is a monomer composed of 12 *α*-helix ribbons (approximately 40%) and eight *β*-sheets (about 11%; **Figure 7C**; **Supplementary Figure S2**) with considerable predicted local similarity (PLS) to the target. Interestingly, the SWISS-model protein structure homology-modeling server confirmed almost the same 3D structure. However, the SWISS-model-based predicted model of SsOAH is a homo-tetramer (sequence identity = 65.78%, sequence similarity = 50%, and coverage = 89%) with a satisfactory global model quality estimate (GMQE = 0.79), QMEANDisCo Global (0.86 ± 0.05), and quaternary structure quality estimate (QSQE = 0.76) (**Figure 7D**; **Supplementary Figure S3**).

### 3.10 _L_-Ornithine down-regulates oxaloacetate acetylhydrolase (*SsOAH*) of *S. sclerotiorum*

**Figure 8:**
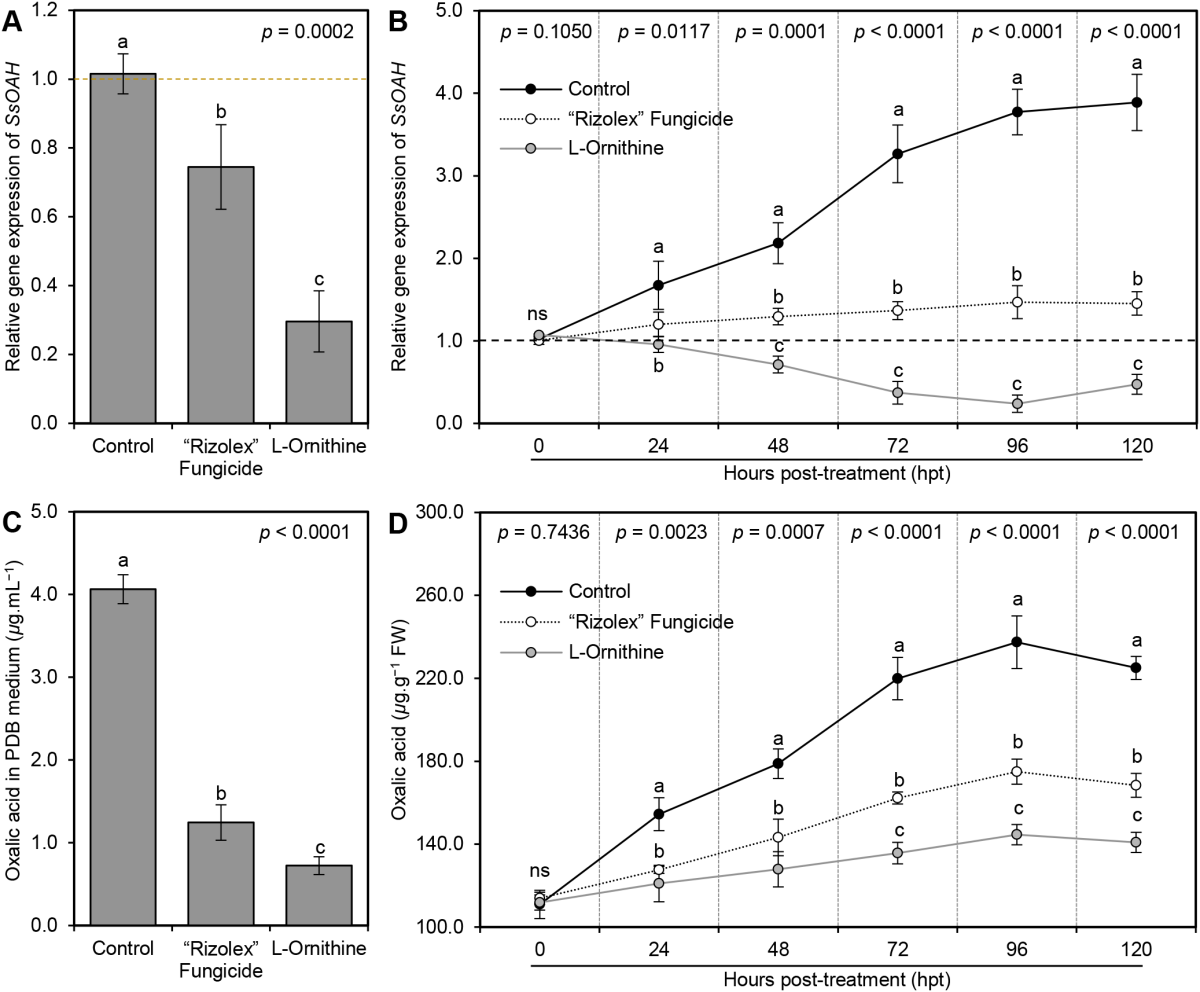
Effect of the exogenous application of _L_-ornithine on the **(A)** oxalic acid secretion in PDB medium (*μ*g.mL^−1^) and oxalic acid content (*μ*g.g^−1^ FW) of *S. sclerotiorum*-infected common beans under greenhouse conditions. Values represent the means ± standard deviation (means ± SD) of five biological replicates (*n* = 5). Different letters indicate statistically significant differences between treatments (*p* < 0.05).

The addition of 40 mg/L _L_-ornithine to the PDB medium significantly down-regulated the gene expression of *SsOAH* in the mycelium of *S. sclerotiorum* (**Figure 8A**). Likewise, although the transcript levels of *SsOAH* were progressively increased in the fungal mycelium collected from non-treated control plants till the end of the experiment (120 hpt), exogenous application of 250 mg/L _L_-ornithine significantly down-regulated the gene expression of *SsOAH* in the fungal mycelia collected from treated plants at 48 hpt and till the end of the experiment (**Figure 8A**).

### 3.11 The diamine _L_-ornithine application diminishes the secretion of oxalic acid

To better understand the relationship between _L_-ornithine and the pathogenicity factor of *S. sclerotiorum*, oxalic acid, the effect of _L_-ornithine application on the secretion of oxalic acid in liquid media (PDB), as well as infected tissues was investigated. Briefly, the addition of 40 mg _L_-ornithine per liter of PDB media significantly reduced the secretion of oxalic acid in the medium (1.24 ± 0.21 *μ*g.mL^−1^) compared to the non-treated control (4.06 ± 0.17 *μ*g.mL^−1^; **Figure 8C**). However, we believe that the observed reduction in oxalic acid levels could be due to the inhibited growth of *S. sclerotiorum* or due to direct inhibition of *SsOAH* activity. Therefore, the fungal mycelium was weighed to normalize oxalic acid production per gram of fungal biomass in our *in vitro* experiments. The results showed that _L_-ornithine (40 mg/L) significantly reduced oxalic acid production even after normalizing fungal biomass, suggesting a direct inhibitory effect on the enzyme’s activity or its expression. Likewise, exogenous application of _L_-ornithine (250 mg/L) significantly reduced the oxalic acid accumulation in *S. sclerotiorum-*infected bean leaves. Although the oxalic acid level in non-treated infected leaves was gradually increased over the time course until it reached its highest peak at 96 hpt (237.40 ± 12.69 *μ*g.g^−1^ FW), _L_-ornithine application significantly lessened the oxalic acid accumulation in treated plants (**Figure 8D**).

## 4 Discussion

The phytopathogenic fungus *S. sclerotiorum* can infect over 600 plant species, with diverse phylogenetic backgrounds (Liang and Rollins, 2018). Therefore, it is noticeably vital to develop novel eco-friendly strategies to manage its spread among agronomically important crops, particularly because the traditional control measures have repeatedly been reported to be inadequate (Cessna et al., 2000). Recently, we proposed two non-proteinogenic amino acids (γ-aminobutyric acid (GABA) and *ß*-alanine) as innovative eco-friendly alternatives to mitigate oxidative stress and enhance the resistance of common bean plants against *S. sclerotiorum*, the causal agent of white mold disease (Nehela et al., 2024). In the current study, we proposed the diamine, _L_-ornithine, as a potential alternative that enhances resilience and boosts the defense mechanisms of common bean plants against *S. sclerotiorum.*_L_-Ornithine application significantly reduced the AUDPC of white mold disease by approximately 78% compared to the non-treated control. It is worth mentioning that _L_-Ornithine application was more efficient in reducing the AUDPC than GABA (74.12%) and *ß*-alanine (67.10) which both were tested in our previous study (Nehela et al., 2024). These findings suggest that non-proteinogenic amino acids (GABA, *ß*-alanine, and _L_-ornithine) might be promising eco-friendly alternatives for white mold disease management for sustainable bean production with a superiority of _L_-ornithine.

Initially, we isolated and characterized four *S. sclerotiorum* isolates. It is worth mentioning that the pathogenicity of these isolates was positively associated with oxalic acid secretion. In other words, the highly virulent isolate #3 of *S. sclerotiorum* was found to secrete more oxalic acid in the PDB media (El-Argawy, 2012). Although the used method in the current study is primarily designed for oxalic acid quantification (Xu and Zhang, 2000), it is possible that other organic acids with similar chemical properties could interfere with the reaction and contribute to the absorbance at 600 nm may result in minor cross-reactivity that cannot be entirely ruled out. Further studies using HPLC or GC-MS-based targeted metabolomics are required to confirm the specificity of oxalic acid and to better understand its role(s) in bean-*S. sclerotiorum* interaction.

The non-specific phytotoxin, oxalic acid, is an essential pathogenicity determinant of *S. sclerotiorum* that is associated with diverse and sometimes contradictory effects on host plants (Marciano et al., 1983; Cessna et al., 2000; Guimarães and Stotz, 2004; Hegedus and Rimmer, 2005; Williams et al., 2011; Heller and Witt-Geiges, 2013; Fagundes-Nacarath et al., 2018; Daniel et al., 2024). Initially, it was proposed to manipulate the pH of the plant cell to create a favorable environment for infection (Marciano et al., 1983; Hegedus and Rimmer, 2005). However, emerging evidence suggests that the negative effects of oxalic acid on host cells are largely independent of both its acidity and its affinity for Ca^2+^ (Cessna et al., 2000) and it might interfere with host defense responses in a more complex manner than previously thought (Cessna et al., 2000; Guimarães and Stotz, 2004; Williams et al., 2011). Although its role in lowering environmental pH and enhancing the activity of cell wall-degrading enzymes (CWDEs) was experimentally proven (Guimarães and Stotz, 2004; Heller and Witt-Geiges, 2013; Daniel et al., 2024), it might play a key contradictory role in reactive oxygen species (ROS) regulation (Cessna et al., 2000; Williams et al., 2011; Daniel et al., 2024). For example, oxalic acid was found to suppress the oxidative burst of the host plant in early infection (Cessna et al., 2000; Williams et al., 2011), while it induces apoptotic-like programmed cell death (PCD) that requires the generation of reactive oxygen species (ROS) in host cells (Williams et al., 2011). In other words, oxalic acid both suppresses and induces host ROS, depending on infection stage, during the compatible interaction (Cessna et al., 2000; Williams et al., 2011).

In agreement with this hypothesis, *S. sclerotiorum*-secreted oxalic acid did not show any phytotoxicity in early infection stages (Heller and Witt-Geiges, 2013) which might be due to the ability of the host cell to catabolize it in the early infection stage, and low concentrations, and translocate it to their vacuoles where it is stored as calcium oxalate (Heller and Witt-Geiges, 2013). However, at late infection stages and upon the accumulation of high oxalic acid levels, it deregulates guard cells and induces foliar wilting (Heller and Witt-Geiges, 2013). Additionally, recent studies proved that oxalic acid is involved in the regulation of fungal endopolygalacturonase (SSPG1) activity at low pH, reinforcing its integral role in *S. sclerotiorum* pathogenesis (Daniel et al., 2024). In addition to its role in virulence, oxalic acid is involved in the mediation of biochemical and physiological changes in the common bean-*Sclerotinia sclerotiorum* interaction, particularly reducing photosynthetic pigments and intensifying oxidative stress (Fagundes-Nacarath et al., 2018). Collectively, these findings highlight oxalic acid as a multifaceted virulence factor, influencing infection dynamics through mechanisms that are still not fully understood, warranting further research into its signaling interference and broader physiological effects.

It is worth mentioning that _L_-ornithine application diminishes the secretion of oxalic acid in PDB media and reduces the oxalic acid accumulation in *S. sclerotiorum-*infected tissues which was associated with a significant reduction in the development and severity of white mold on common bean plants under greenhouse conditions. We understand that the reduced oxalic acid levels in PDB media or within infected tissue might be due to the reduced mycelial mass of *S. sclerotiorum* since _L_-ornithine showed strong antifungal activity against the fungal pathogen and significantly reduced the mycelial growth.

Moreover, the role of polyamine, as well as non-proteinogenic amino acids, during *S. sclerotiorum* infection has gained increasing attention, particularly in the context of oxalic acid production and fungal development (Walker et al., 2023; Nehela et al., 2024). Recently, it was proven that silencing of *Abhydrolase*-3 from *S. sclerotiorum* via host-induced gene silencing (HIGS) in transgenic *Arabidopsis thaliana* (AT1703) significantly downregulated the expression of key polyamine biosynthesis genes, including *Ornithine decarboxylase (ODC)*, *Spermine synthase*, and *Spermidine synthase* of the fungal pathogen and ultimately slowed fungal infection on AT1703 plants compared to their wild type ones (Walker et al., 2023). Our study aligns with and expands on these findings by showing that the exogenous application of _L_-ornithine significantly suppressed the mycelial growth of *S. sclerotiorum* and reduced the production of oxalic acid, which both were correlated with a significant reduction in disease severity in common bean. ODC is a rate-limiting enzyme in polyamine biosynthesis that catalyzes the decarboxylation of ornithine to produce putrescine, the first step in polyamine synthesis (Michael, 2016). We suggest that _L_-ornithine might interfere with this pathway in the causal agent and negatively affect fungal development. The observed *in vitro* antifungal activity of _L_-ornithine may, therefore, be linked to its interference with the polyamine biosynthesis pathway in *S. sclerotiorum*. Taken together, these findings suggest a novel approach for managing white mold through metabolic disruption of *S. sclerotiorum* via polyamine pathway interference. However, further studies are required to explore the _L_-ornithine- *S. sclerotiorum*-oxalic acid interactions as a potential disease control strategy.

It was reported previously that the oxaloacetate acetylhydrolase gene of *S. sclerotiorum* plays a vital role in the fungal oxalic acid accumulation and virulence (Rana et al., 2022) and its deletion mutants failed to accumulate oxalic acid in culture or during plant infection, exhibiting reduced pathogenicity, and produce limited lesions on host plants (Liang et al., 2015; Rana et al., 2022) highlighting the key role of oxalic acid in the disease development. In the current study, *in silico* analysis showed that the *S. sclerotiorum* genome possesses a putative oxaloacetate acetylhydrolase (SsOAH) protein that is highly similar in its functional analysis, conserved domains, and topology to the OAH protein from *A. fijiensis* and *P. lagena*. It is worth mentioning that the addition of _L_-ornithine to the PDB medium down-regulated the gene expression of *SsOAH* in the mycelium of *S. sclerotiorum*. Moreover, our findings showed that exogenous application of _L_-ornithine can effectively down-regulate *SsOAH* expression both *in vitro* and in infected plant tissues, leading to a reduction in the accumulation of oxalic acid and a corresponding decrease in disease severity. It was reported previously that targeting a gene encoding oxaloacetate acetylhydrolase (*Ssoah1*) of *S. sclerotiorum* via HIGS resulted in an oxalic acid deficiency and a non-pathogenic interaction on soybean (McCaghey et al., 2021). The current study builds on previous work and investigates the potential role of *SsOAH* in *S. sclerotiorum* virulence, particularly with oxalic acid production. We believe that manipulating the production of oxalic acid, either through silencing *SsOAH* (McCaghey et al., 2021) or inhibiting its activity with compounds like _L_-ornithine, can serve as an effective strategy for managing *S. sclerotiorum* infections. Moreover, both findings of the current investigation and previous studies highlight the importance of targeting oxalic acid biosynthesis pathway as a promising approach to control *S. sclerotiorum*.

Although current data does not fully differentiate the direct causes of the observed reduction in oxalic acid levels. However, our findings strongly suggest that multiple factors could contribute to this reduction. For instance, *in vitro* antifungal assays demonstrated that _L_-ornithine significantly inhibits the radial mycelial growth of *S. sclerotiorum*, which likely reduces the overall metabolic activity of the pathogen, including oxalic acid production. Moreover, gene expression analysis showed that _L_-ornithine downregulated *SsOAH* transcript levels in both *in vitro* cultures and infected plant tissues, suggesting that reduced oxalic acid production is at least partially due to transcriptional suppression of this key enzyme. Additionally, the observed reduction in oxalic acid levels, even when normalized to fungal biomass, suggests that _L_-ornithine may directly or indirectly inhibit the enzymatic activity of *SsOAH*. However, further studies are required to confirm this hypothesis.

Likewise, oxalic acid-deficient mutants of *S. sclerotiorum* were nonpathogenic while the oxalic acid-producing wild type was pathogenic to whole plants, stems, and leaves under growth chamber conditions (Godoy et al., 1990). Moreover, the fungal oxalic acid-deficient mutants were nonpathogenic, and apoptotic-like features were not observed following plant inoculation (Kim et al., 2008) and resulted in restricted growth, reminiscent of an HR-like response (Williams et al., 2011). Herein, our findings showed that _L_-ornithine application significantly down-regulated *SsOAH*, diminished the secretion of oxalic acid, and inhibited the growth of *S. sclerotiorum* to reduce its pathogenicity in treated plants in contrast to non-treated ones which had higher oxalic acid levels and showed overwhelming disease and runaway cell death. Meanwhile, _L_-ornithine stimulated the growth of *S. sclerotiorum*-infected bean plants as expressed by the plant height, number of branches, and number of leaves per plant suggesting no phytotoxicity on the treated bean plants. It was reported previously that the amine functional group of ornithine and its derivative *N*-acetylornithine play a key role in reducing the toxicity of fumonisins (a mycotoxin produced by numerous fungal species such as *Fusarium* sp. and *Aspergillus* sp.) (Renaud et al., 2021).

Although the specific biochemical, physiological, and molecular mechanisms responsible for the protective role of _L_-ornithine are not fully understood, several potential mechanisms have been proposed. An acceptable hypothesis that could explain how _L_-ornithine effectively enhances the resilience of common bean plants against *S. sclerotiorum* is due to its ability to mitigate oxidative stress and modulate the redox status within infected plants. Exogenous ornithine was reported previously to improve the plant response to abiotic stress such as drought (Hussein et al., 2019) and salinity (da Rocha et al., 2012) via contribution to proline accumulation (da Rocha et al., 2012) or modulation of oxidative stress (Hussein et al., 2019).

Our findings showed that infection with *S. sclerotiorum* induced oxidative stress in infected bean plants, leading to the overproduction of ROS such as H_2_O_2_ and O_2_^•−^, as well as lipid peroxidation which might contribute to disease progression (Torres et al., 2006). Nevertheless, exogenous application of _L_-ornithine successfully decreases ROS levels and alleviates oxidative stress within *S. sclerotiorum*-infected plants via the induction of multilayered antioxidant defense machinery. The complex antioxidant defense contains two key mechanisms, enzymatic and non-enzymatic antioxidant defense machinery (Sharma et al., 2022). The enzymatic antioxidants act as the front line in antioxidant defenses, however, the non-enzymatic antioxidants characterize the second line of defense against ROS (Sharma et al., 2022).

In the current study, _L_-ornithine boosts the non-enzymatic (total soluble phenolics and flavonoids) as well as the enzymatic (CAT, POX, PPO, *PvCAT1*, *PvSOD*, and *PvGR*) antioxidant defense machinery of *S. sclerotiorum*-infected bean plants. In agreement with these findings, _D_-ornithine increased the activity of major antioxidant enzymes (SOD, CAT, and POX) in tobacco cells under salinity (Ghahremani et al., 2013) and enhanced the activity of CAT and POX in sugar beet plants under drought stress (Hussein et al., 2019). Similarly, L-ornithine slightly increased the POX (at 48 hpi) and CAT activity (at 72 hpi), but not phenylalanine ammonia-lyase (PAL) in lime (*Citrus aurantifolia*) plants infected with the bacterial phytopathogen *Xanthomonas citri* subsp. *citri*, the causal agent of Citrus bacterial canker (Hasabi et al., 2014). Collectively, these findings demonstrate that _L_-ornithine substantially enhances both the enzymatic and non-enzymatic antioxidant defense machinery of bean plants to reduce ROS levels and maintain their homeostasis to ease the undesirable oxidative stress caused by *S. sclerotiorum*.

Another hypothesis that might explain the potential role(s) of _L_-ornithine against *S. sclerotiorum* is due to its contribution to amino acids and proline accumulation. Exogenous ornithine effectively contributes to proline accumulation in cashew leaves under salt stress (da Rocha et al., 2012). Moreover, higher levels of proline were accumulated in *Arabidopsis thaliana* during defense against pathogens (Monteoliva et al., 2014) and proline catabolism usually enhanced during the early stages of pathogen infection. Likewise, the proline intermediate pyrroline-5-carboxylate (P5C) might contribute to plant defense against invading pathogens.

The hypothesis that _L_-ornithine might be involved in the common bean defense responses against *S. sclerotiorum* by contributing proline and P5C accumulation is supported by several experimental evidence on proline metabolism under stress (Qamar et al., 2015; Rizzi et al., 2015). For instance, it was suggested previously that P5CDH contributes to the modulation of the proline biosynthesis pathway and its catabolism after the activation of ProDH in response to biotic and abiotic stressors (Rizzi et al., 2015). The balance between the biosynthetic and catabolic pathways is key to maintaining cellular homeostasis. Likewise, P5C was reported to play a key role in plant defense against invading pathogens (Qamar et al., 2015) where it triggers a hypersensitive response (HR) and contributes to the induction of ROS. Collectively, these findings suggest that the proline-P5C pathway, as well as its regulatory enzymes such as P5CDH and ProDH, might be involved in the activation of defense responses such as HR and ROS accumulation after pathogen attack. In the context of our study, the exogenous application of _L_-ornithine might boost proline levels by inducing P5C accumulation and potentially enhancing the plant’s defense against *S. sclerotiorum* infection through the modulation of HR-like responses and ROS production. Accordingly, the regulation of proline and P5C metabolism by _L_-ornithine supplementation represents a complementary mechanism by which plants combat pathogen infection, in addition to the above-discussed impact on oxalic acid production.

It is worth noting that the P5C dehydrogenase (P5CDH) mutant of *A. thaliana* showed higher proline dehydrogenase (*ProDH*) and ornithine aminotransferase (OAT) activity, as well as higher levels of ornithine and proline when challenged with exogenous proline, or infection with the bacterial pathogen *Pseudomonas syringae* pv. *tomato* (Monteoliva et al., 2014). Activation of OAT and higher ornithine and proline levels proposes that ornithine is a precursor for proline biosynthesis (Monteoliva et al., 2014) and contributes to multiple abiotic stress tolerances. Additionally, proline plays a key role in redox homeostasis and energy transfer reactions, which lead to plant resistance against pathogens or programmed cell death (PCD) (Anwar et al., 2018). Interestingly, our findings showed that exogenous _L_-ornithine efficiently enhanced the total amino acids and proline content of *S. sclerotiorum*-infected bean plants. Nevertheless, while the spectrophotometric approach provides a reliable estimate of total free amino acids, advanced techniques such as LC/MS could complement these findings by offering detailed amino acid profiles and insights into non-enzymatic antioxidant contributions.

## 5 Conclusion

**Figure 9:**
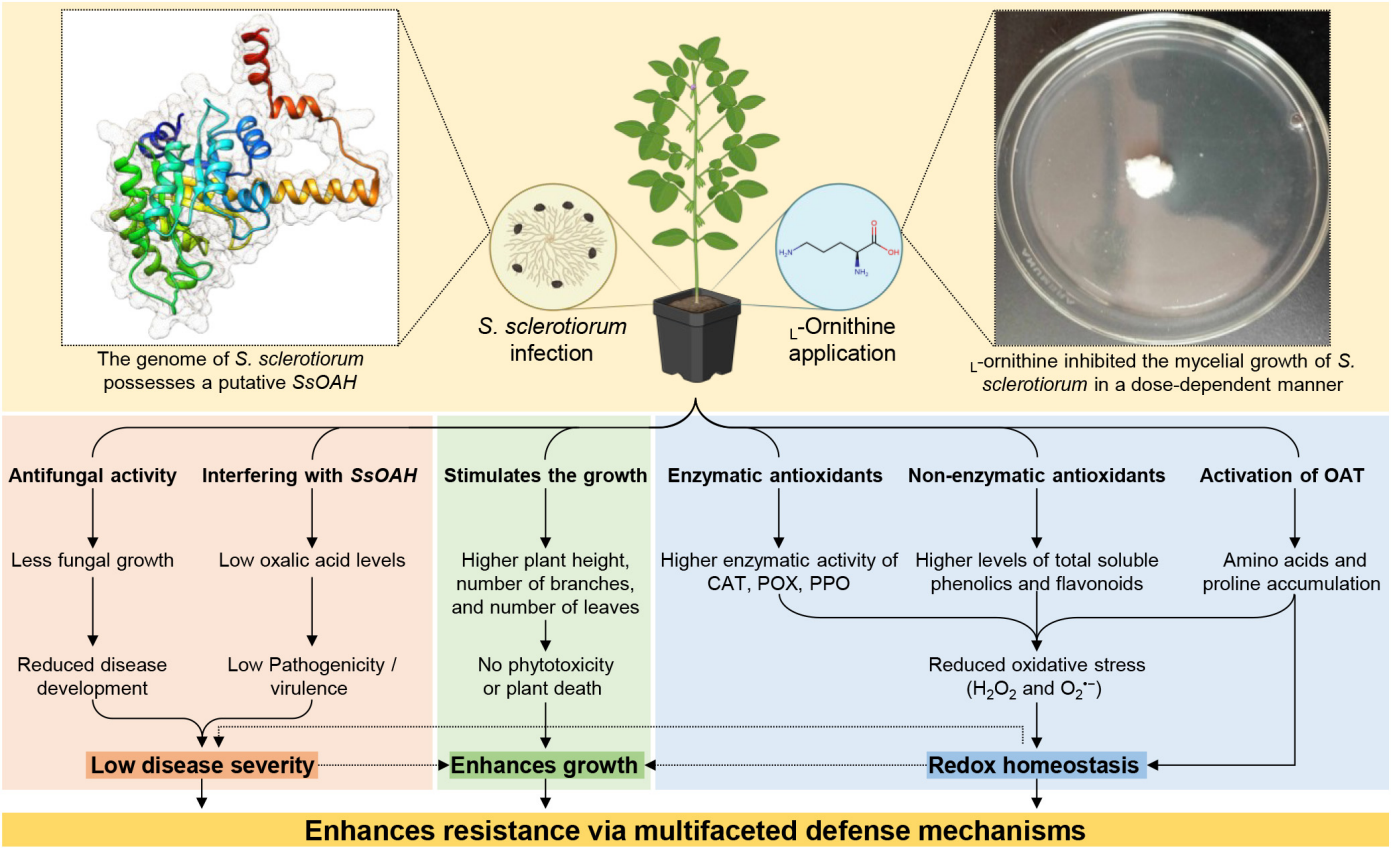
Hypothetical model for the potential effects of _L_-ornithine supplementation on the growth and response of common beans against *S. sclerotiorum*, the causal agent of white mold disease under greenhouse conditions. In this model, we suggest that _L_-ornithine supplementation via seed priming combined with root drench might enhance the response of common bean plants to the infection with the soil-borne fungal pathogen, *S. sclerotiorum*, via modulation of multilayered defense machinery. Briefly, (i) _L_-Ornithine showed antifungal activity and progressively inhibited the mycelial growth of *S. sclerotiorum* in a dose-dependent manner. Moreover, _L_-ornithine supplementation down-regulates oxaloacetate acetylhydrolase (*SsOAH*) of *S. sclerotiorum* in the PDB medium and the fungal mycelia collected from treated plants at 48 hpt. Together, the antifungal activity of _L_-ornithine and interfering with *SsOAH* diminished the development of white mold on common bean plants under greenhouse conditions. (ii) _L_-Ornithine supplementation progressively enhanced the plant height, number of branches per plant, and number of leaves per plant throughout 42 dpi. Collectively, these findings suggest that _L_-ornithine has no phytotoxicity on the treated bean plants, but it even stimulates their growth. (iii) _L_-Ornithine mitigated the oxidative stress of *S. sclerotiorum*-infected bean plants via the induction of multilayered antioxidant defense machinery including enzymatic (CAT, POX, PPO, *PvCAT1*, *PvSOD*, and *PvGR*) and non-enzymatic (total soluble phenolics and flavonoids) antioxidants, as well as total amino acids and proline accumulation. Collectively, these findings suggest that _L_-ornithine supplementation induces multilayered antioxidant defense machinery to maintain the Redox homeostasis within *S. sclerotiorum*-infected common bean plants.

In conclusion, our findings suggest that _L_-ornithine might be a promising eco-friendly alternative in management programs for white mold disease caused by *S. sclerotiorum* in common beans. _L_-Ornithine exhibited a strong antifungal *in vitro* and progressively reduced the development of white mold on common bean plants under greenhouse conditions. These fungistatic actions were combined with the ability of _L_-ornithine to mitigate the oxidative stress of *S. sclerotiorum*-infected bean plants via the activation of multilayered antioxidant defense machinery (enzymatic and non-enzymatic antioxidants), as well as proline accumulation (**Figure 9**). Moreover, _L_-ornithine did not show any phytotoxicity on treated plants but enhanced their growth. Collectively, all these findings establish _L_-ornithine as a promising eco-friendly alternative for sustainable bean production. However, further investigations are required to optimize the application strategies for _L_-ornithine use in the field and to explore their potential in integrated pest management programs. Moreover, further studies are required to better understand the impact on bean production, including potential effects on the number of pods, size of beans, and total yield. While our current study focuses on the antifungal activity and plant response, we acknowledge the importance of evaluating agronomic traits. Likewise, investigating the effect(s) of _L_-ornithine supplementation on healthy (non-infected) bean plants is another direction that requires further research, including its potential impact on growth, performance, physiological responses, and yield. This additional research might provide a more comprehensive understanding of the broader implications of _L_-ornithine in bean physiology particularly and other legumes in general.

## Data Availability

The original contributions presented in the study are included in the article/**Supplementary Material**. Further inquiries can be directed to the corresponding authors.
