# Supplementary material for: Ornithine enhances common bean growth and defense against white mold disease via interfering with SsOAH and diminishing the biosynthesis of oxalic acid in Sclerotinia sclerotiorum
Source: Front Plant Sci. 2025 Apr 4;16:1483417. doi: 10.3389/fpls.2025.1483417 (PMC12006162; doi:10.3389/fpls.2025.1483417)
Supplement: Supplementary file 1 [file DataSheet1.docx]

Supplementary Material

Ornithine Enhances Common Bean growth and Defense against White Mold Disease via Interfering with *SsOAH* and Diminishing the Biosynthesis of Oxalic acid in *Sclerotinia sclerotiorum*

Yasser Nehela ^1,*^, Yasser S. A. Mazrou ^2^, Nehad A. EL_Gammal ^3^, Osama Atallah ^4^, Abdelrazek S. Abdelrhim ^5^, Sumit Kumar ^6^, Temoor Ahmed ^7^, Qurban Ali ^8^, Abeer H. Makhlouf ^8^ and Warda A. M. Hussain ^3^

^1^ Department of Agricultural Botany, Faculty of Agriculture, Tanta University, Tanta 31527, Egypt.

^2^ Business Administration Department, Community College, King Khalid University, Guraiger, 62529, KSA.

^3^ Plant Pathology Research Institute, Agricultural Research Center, Giza, 12619, Egypt.

^4^ Department of Plant Pathology, Faculty of Agriculture, Zagazig University, Zagazig, Egypt.

^5^ Department of Plant Pathology, Faculty of Agriculture, Minia University, Minia 85721, Egypt.

^6^ Institute of Agricultural Sciences, Banaras Hindu University, Varanasi, Uttar Pradesh 221005, India

^7^ Institute of Biotechnology, Zhejiang University, Hangzhou, China

^8^ Department of Biology, College of Science, United Arab Emirates University, Al-ain, Abu-Dhabi, UEA.

^9^ Department of Agricultural Botany, Faculty of Agriculture, Minufiya University, Shebeen El-Kom, Egypt.

*** Correspondence:**

**Yasser Nehela :** [yasser.nehela@agr.tanta.edu.eg](mailto:yasser.nehela@agr.tanta.edu.eg)

**Qurban Ali:** [rattarqurban@uaeu.ac.ae](mailto:rattarqurban@uaeu.ac.ae)

Keywords: Ornithine; white mold; bean; antioxidant; *Sclerotinia*; *OAH*; Oxalic acid

# Supplementary Figures


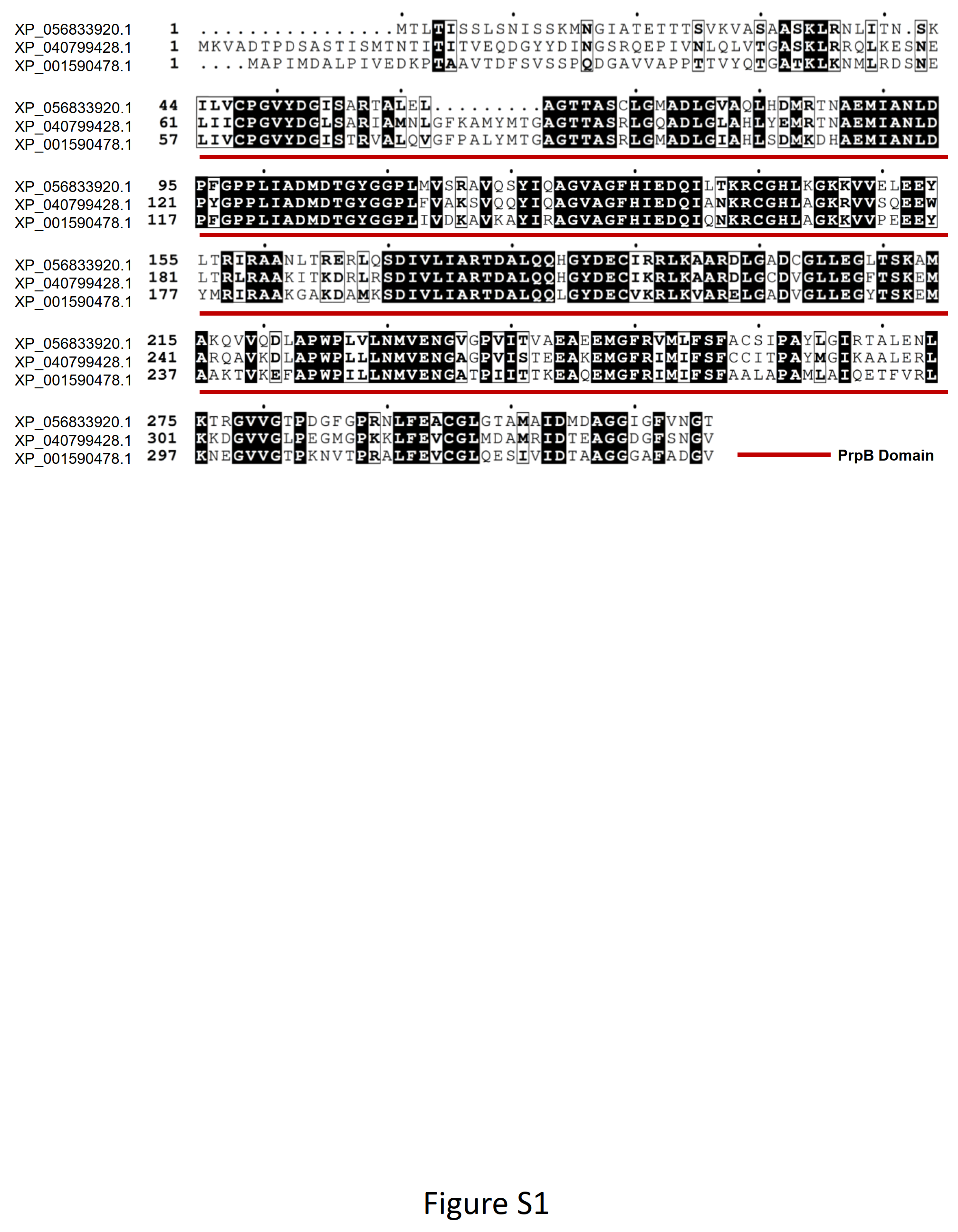


**Figure S1.** Multiple sequence alignment of the top matched AA sequence of putative oxaloacetate acetylhydrolase (*SsOAH*) protein from *S. sclerotiorum* (GenBank accession no. [XP_001590478.1](https://www.ncbi.nlm.nih.gov/protein/XP_001590478.1?report=genbank&log$=prottop&blast_rank=1&RID=4EN0WPUR016), 338 aa) with OAH from *Aspergillus fijiensis* CBS 313.89 (*AfOAH*; GenBank Accession No. [XP_040799428.1](https://www.ncbi.nlm.nih.gov/protein/XP_040799428.1); 342 aa) and *Penicillium lagena* (*PlOAH*; GenBank Accession No. [XP_056833920.1](https://www.ncbi.nlm.nih.gov/protein/XP_056833920.1); 316 aa). Conserved amino acids are shown with black shading whereas highly similar residues are shaded in gray. The listed genes were identified based on available in GenBank, the National Center for Biotechnology Information website (NCBI, <http://www.ncbi.nlm.nih.gov/gene/>). PrpB conserved domain is underlined with a red-colored line.


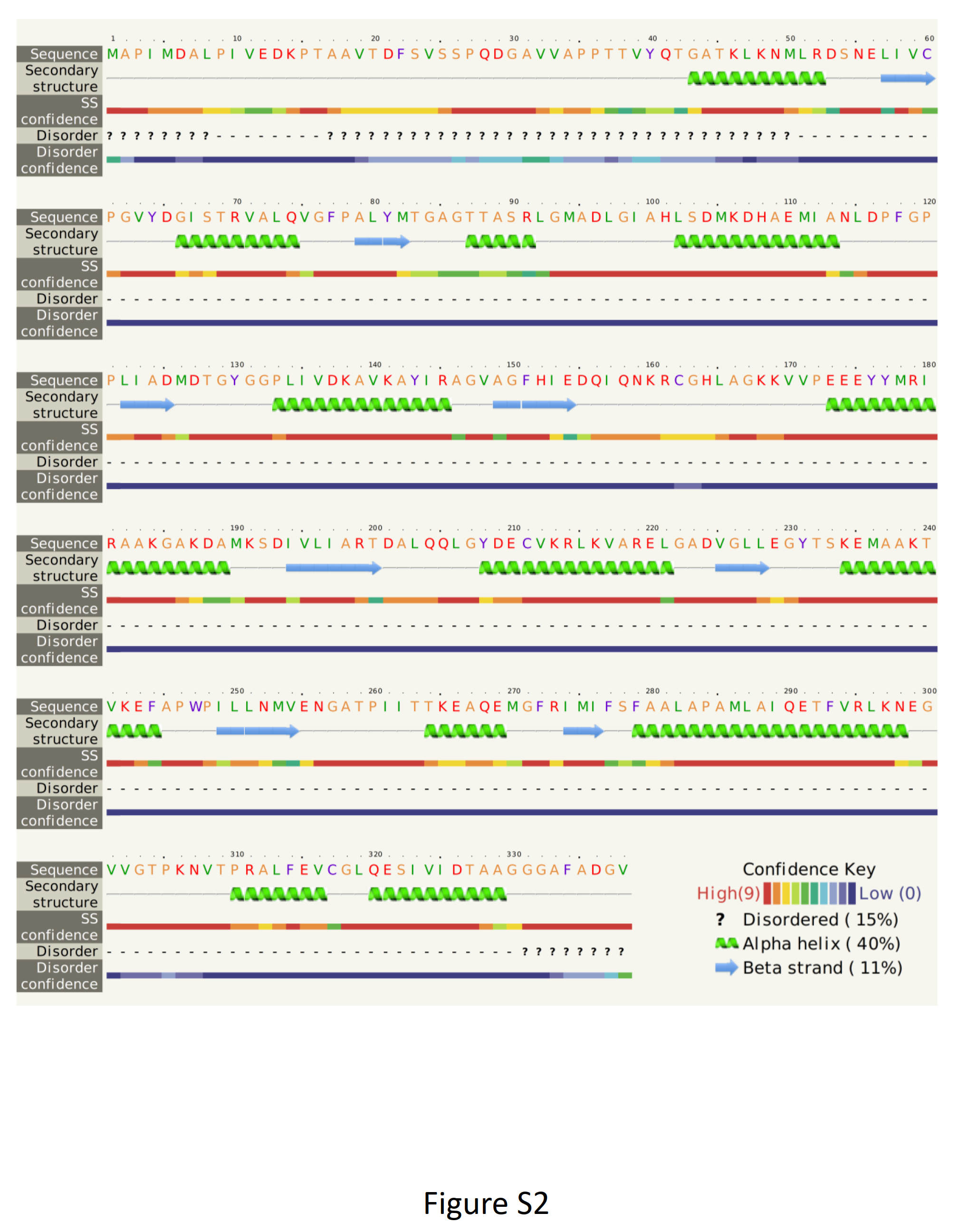


**Figure S2.** Model–template alignment of putative oxaloacetate acetylhydrolase (*SsOAH*) protein from *S. sclerotiorum* (GenBank accession no. [XP_001590478.1](https://www.ncbi.nlm.nih.gov/protein/XP_001590478.1?report=genbank&log$=prottop&blast_rank=1&RID=4EN0WPUR016), 338 aa) with the crystal structure of oxaloacetate acetylhydrolase in the protein data bank (PDB ID: [3lye.1.A](https://swissmodel.expasy.org/templates/3lye.1)) as predicted by Phyre2 tool. *α*-helices of the predicted secondary structures are shown as green spirals, whereas *β*-sheets are represented by light-blue arrows. The confidence key is presented at the bottom right corner of the figure.


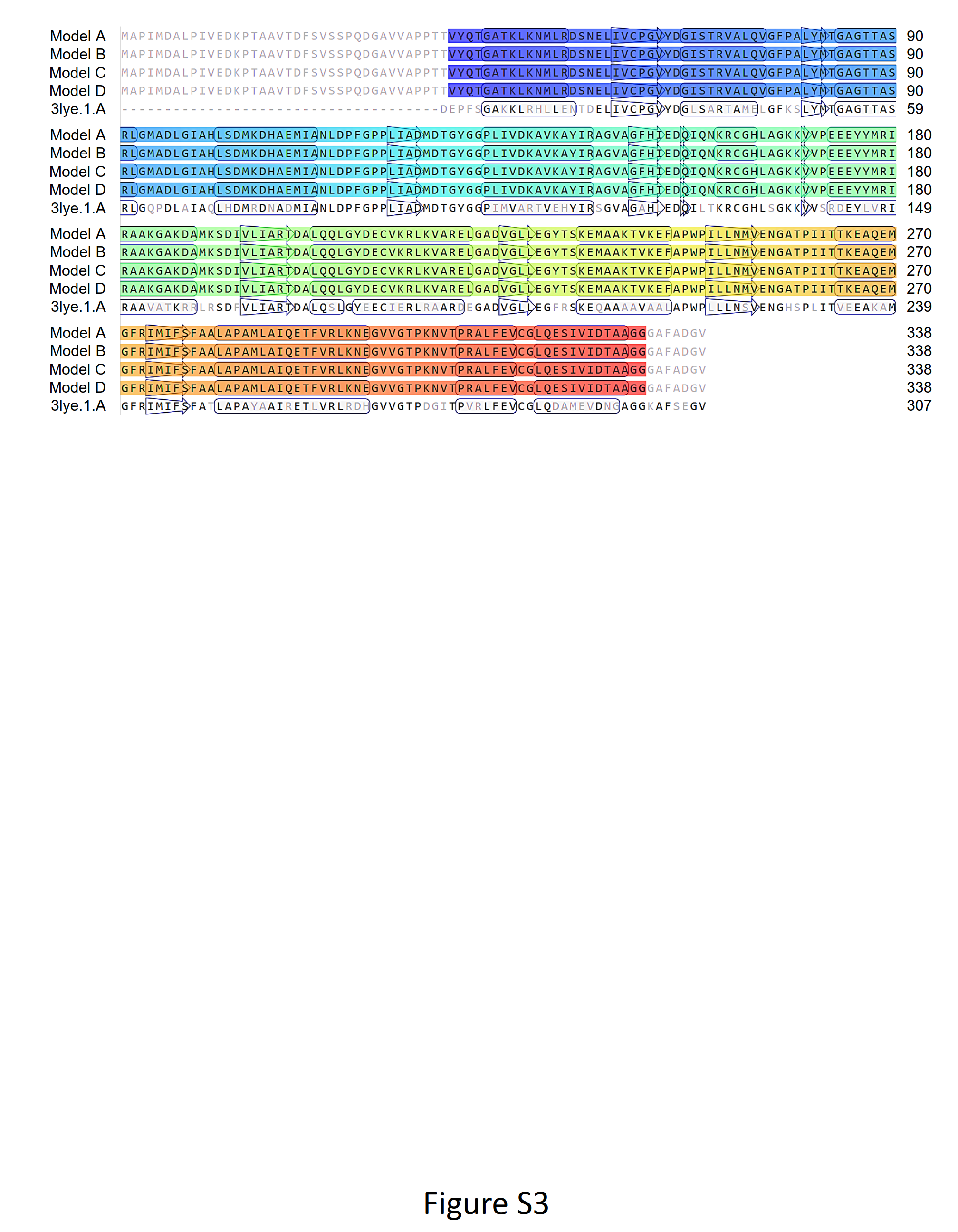


**Figure S3.** Model–template alignment of putative oxaloacetate acetylhydrolase (*SsOAH*) protein from *S. sclerotiorum* (GenBank accession no. [XP_001590478.1](https://www.ncbi.nlm.nih.gov/protein/XP_001590478.1?report=genbank&log$=prottop&blast_rank=1&RID=4EN0WPUR016), 338 aa) with the crystal structure of oxaloacetate acetylhydrolase in the protein data bank (PDB ID: [3lye.1.A](https://swissmodel.expasy.org/templates/3lye.1)) as generated by the SWISS-MODEL server. AA sequences of each model were aligned with the template ([4dji.1.A](https://swissmodel.expasy.org/templates/4dji.1)). *α*-helices of the predicted secondary structures are shown as rectangles, whereas *β*-sheets are represented by arrows. Matched sequences are black-colored.

# Supplementary Tables

**Table S1.** *Sclerotinia* strains/isolates that produce significant alignments with *S. sclerotiorum* - Isolate #3 retrieved from the recently available data in NCBI GenBank ^a^.

| **Isolate/strain** | **Accession No.** | **Location** | **Host** | **Year** | **Max Score** | **Total Score** | **Query Cover (%)** | **E value** | **Identity (%)** | **Accession Length** |
| --- | --- | --- | --- | --- | --- | --- | --- | --- | --- | --- |
| *S. sclerotiorum* | [MK074848.1](https://www.ncbi.nlm.nih.gov/nucleotide/MK074848.1?report=genbank&log$=nucltop&blast_rank=1&RID=W8MTS7Z3013) | India: Delhi | Pulse crops | 2010 | 979 | 979 | 100 | 0 | 99.81 | 543 |
| *S. sclerotiorum* Isolate PSP-1 | [MN216247.1](https://www.ncbi.nlm.nih.gov/nucleotide/MN216247.1?report=genbank&log$=nucltop&blast_rank=2&RID=W8MTS7Z3013) | Bangladesh | pea | 2019 | 979 | 979 | 100 | 0 | 99.81 | 572 |
| *S. sclerotiorum* Isolate LPM36 | [MK896659.1](https://www.ncbi.nlm.nih.gov/nucleotide/MK896659.1?report=genbank&log$=nucltop&blast_rank=3&RID=W8MTS7Z3013) | USA | Dry pea seed | 2019 | 979 | 979 | 100 | 0 | 99.81 | 540 |
| *S. sclerotiorum* Voucher CSS2 | [MK828199.1](https://www.ncbi.nlm.nih.gov/nucleotide/MK828199.1?report=genbank&log$=nucltop&blast_rank=4&RID=W8MTS7Z3013) | India | Chickpea | 2019 | 979 | 979 | 100 | 0 | 99.81 | 558 |
| *S. sclerotiorum* Isolate 21KTOPS1 | [PQ651498.1](https://www.ncbi.nlm.nih.gov/nucleotide/PQ651498.1?report=genbank&log$=nucltop&blast_rank=5&RID=W8MTS7Z3013) | Russia | Jerusalem artichoke | 2021 | 979 | 979 | 100 | 0 | 99.81 | 553 |
| *S. matthiolae* strain CBS 111.17 | [MH854660.1](https://www.ncbi.nlm.nih.gov/nucleotide/MH854660.1?report=genbank&log$=nucltop&blast_rank=6&RID=W8MTS7Z3013) | Switzerland | - | 2017 | 979 | 979 | 100 | 0 | 99.81 | 546 |
| *S. sclerotiorum* Isolate SS1 | [OR623245.1](https://www.ncbi.nlm.nih.gov/nucleotide/OR623245.1?report=genbank&log$=nucltop&blast_rank=7&RID=W8MTS7Z3013) | India | Chickpea | 2022 | 979 | 979 | 100 | 0 | 99.81 | 544 |
| *S. sclerotiorum* Isolate COLPAT-586 | [MH318571.1](https://www.ncbi.nlm.nih.gov/nucleotide/MH318571.1?report=genbank&log$=nucltop&blast_rank=8&RID=W8MTS7Z3013) | Spain | Rosemary | 2018 | 979 | 979 | 100 | 0 | 99.81 | 564 |
| *S. sclerotiorum* Strain EPSS-9 | [MH393290.1](https://www.ncbi.nlm.nih.gov/nucleotide/MH393290.1?report=genbank&log$=nucltop&blast_rank=9&RID=W8MTS7Z3013) | China | - | 2018 | 979 | 979 | 100 | 0 | 99.81 | 611 |
| *S. sclerotiorum* Strain EPSS-1 | [MH393289.1](https://www.ncbi.nlm.nih.gov/nucleotide/MH393289.1?report=genbank&log$=nucltop&blast_rank=10&RID=W8MTS7Z3013) | China | - | 2018 | 979 | 979 | 100 | 0 | 99.81 | 614 |
| *S. sclerotiorum* Voucher J2 | [MH298771.1](https://www.ncbi.nlm.nih.gov/nucleotide/MH298771.1?report=genbank&log$=nucltop&blast_rank=11&RID=W8MTS7Z3013) | China | Sunflower | 2015 | 979 | 979 | 100 | 0 | 99.81 | 541 |
| *S. sclerotiorum* Voucher HR1 | [MH298770.1](https://www.ncbi.nlm.nih.gov/nucleotide/MH298770.1?report=genbank&log$=nucltop&blast_rank=12&RID=W8MTS7Z3013) | China | Sunflower | 2015 | 979 | 979 | 100 | 0 | 99.81 | 541 |
| *S. sclerotiorum* Voucher D1 | [MH298768.1](https://www.ncbi.nlm.nih.gov/nucleotide/MH298768.1?report=genbank&log$=nucltop&blast_rank=13&RID=W8MTS7Z3013) | China | Sunflower | 2015 | 979 | 979 | 100 | 0 | 99.81 | 542 |
| *S. sclerotiorum* strain ICMP 11691 | [PQ214219.1](https://www.ncbi.nlm.nih.gov/nucleotide/PQ214219.1?report=genbank&log$=nucltop&blast_rank=14&RID=W8MTS7Z3013) | New Zealand | Soft speargrass | 2024 | 979 | 979 | 100 | 0 | 99.81 | 578 |
| *S. sclerotiorum* isolate SSC2JHU | [MG249967.1](https://www.ncbi.nlm.nih.gov/nucleotide/MG249967.1?report=genbank&log$=nucltop&blast_rank=15&RID=W8MTS7Z3013) | USA | Mexican cotton | 2017 | 979 | 979 | 100 | 0 | 99.81 | 565 |
| *S. sclerotiorum* strain CN144F7 | [OP596170.1](https://www.ncbi.nlm.nih.gov/nucleotide/OP596170.1?report=genbank&log$=nucltop&blast_rank=39&RID=W8MTS7Z3013) | South Africa | Soybean | 2022 | 979 | 979 | 100 | 0 | 99.81 | 812 |
| *S. sclerotiorum* isolate YKY211 | [OR206374.1](https://www.ncbi.nlm.nih.gov/nucleotide/OR206374.1?report=genbank&log$=nucltop&blast_rank=72&RID=W8MTS7Z3013) | China | Stock | 2021 | 979 | 979 | 100 | 0 | 99.81 | 548 |
| *S. sclerotiorum* isolate BL31 | [OQ891472.1](https://www.ncbi.nlm.nih.gov/nucleotide/OQ891472.1?report=genbank&log$=nucltop&blast_rank=73&RID=W8MTS7Z3013) | India | Berseem clover | 2021 | 979 | 979 | 100 | 0 | 99.81 | 575 |
| *S. sclerotiorum* isolate SFJ1 | [ON651665.1](https://www.ncbi.nlm.nih.gov/nucleotide/ON651665.1?report=genbank&log$=nucltop&blast_rank=77&RID=W8MTS7Z3013) | China | Milk thistle | 2022 | 979 | 979 | 100 | 0 | 99.81 | 583 |
| *S. sclerotiorum* strain AUMC 15575 | [OP164563.1](https://www.ncbi.nlm.nih.gov/nucleotide/OP164563.1?report=genbank&log$=nucltop&blast_rank=1&RID=W8K07174016) | Egypt | Pot marigold | 2022 | 979 | 979 | 100 | 0 | 99.81 | 525 |

^a^ The listed accessions were identified using the Nucleotide-Nucleotide BLAST (BLASTn) using *S. sclerotiorum* - Isolate #3 (GenBank accession no. PV202792) as a query sequence against recently available in GenBank, the National Center for Biotechnology Information database (NCBI, <http://www.ncbi.nlm.nih.gov/gene/>), using the compositionally adjusted substitution matrices (Altschul et al., 2005).

**Table S2: Modified Petzoldt and Dickson rating scale for the white mold of common bean ^a^.**

| **Score** | **Description** |
| --- | --- |
| 1 | No sign of stem/branch infection adjacent to the inoculant. |
| 2 | Stem/branch infected but the invasion of the first internode <1 inch. |
| 3 | Stem/branch invasion of the first internode >1 inch but not reached the first node. |
| 4 | Stem/branch invasion reached the first node, but no further. |
| 5 | Stem/branch invasion passed the first node, but the invasion of the second internode <1 inch. |
| 6 | Stem/branch invasion of the second internode >1 inch but not reached the second node. |
| 7 | Stem/branch invasion reached the second node, but no further. |
| 8 | Stem/branch invasion passed the second node, but the invasion of the third internode <1 inch. |
| 9 | Stem/branch invasion of the third internode >1 inch leading to plant death. |

^a^ Adapted from (Petzoldt and Dickson, 1996) and modified by (Terán et al., 2006).

**Table S3:** Primers sequences for quantitative RT-PCR of the antioxidant-associated genes of common bean (*Phaseolus vulgaris*) and putative oxaloacetate acetylhydrolase (*SsOAH*) from *S. sclerotiorum*.

| **Gene name** | **Reference Sequence** |  | **5′ - 3′ primer sequence** | **Tm** | **Product size** |
| --- | --- | --- | --- | --- | --- |
| **Antioxidant-associated genes of common bean (*Phaseolus vulgaris*)** | | | | | |
| *PvSOD* | XM_068639556.1 | F: | CTGAGGATGAGAGTCGCCAT | 58.96 | 183 |
|  |  | R: | TGCTAAGCTCATGACCACCT | 58.72 |  |
| *PvCAT1* | KF033307.1 | F: | CAACCATAGCCATGCCACTC | 58.97 | 155 |
|  |  | R: | TAATGTCCTCGGGCCAAGTT | 59.01 |  |
| *PvGR* | KY195009.1 | F: | ACAAAGGTACAGTGGACGGT | 58.87 | 177 |
|  |  | R: | TCCCAAATTCCTCACCTCGA | 58.35 |  |
| *PvActin-3* | XM_068616709.1 | F: | CAGAGCGGGAAATTGTGAGG | 58.91 | 157 |
|  |  | R: | CGGAATCGTTCAGCACCAAT | 58.91 |  |
| **Putative oxaloacetate acetylhydrolase (*SsOAH*) from *S. sclerotiorum*.** | | | | | |
| *SsOAH* | XM_001590428.1 | F: | GGTGCCAAAGATGCCATGAA | 59.10 | 187 |
|  |  | R: | ATGGGGCGAACTCCTTAACA | 59.01 |  |
| *SsActin* | XM_001589919.1 | F: | TCTCACTTCCTCACGCCATT | 59.02 | 159 |
|  |  | R: | CAAGAGCGACGTAACAGAGC | 59.01 |  |

*PvSOD*: PREDICTED: Phaseolus vulgaris superoxide dismutase [Cu-Zn] (LOC137831752)

*PvCAT*: Peroxisomal catalase from *Phaseolus vulgaris*

*PvGR*: Glutathione reductase from *Phaseolus vulgaris*

*PvActin-3:* PREDICTED: *Phaseolus vulgaris* actin-3 (LOC137814134), transcript variant X1

*SsOAH:* Oxaloacetate acetylhydrolase from *S. sclerotiorum*

*SsActin*: Actin from *Sclerotinia sclerotiorum* 1980 UF-70

Table S4. Sequences from *Sclerotinia sclerotiorum* that produce significant alignment with oxaloacetate acetylhydrolase (OAH) protein from *Aspergillus fijiensis* CBS 313.89 and *Penicillium lagena* ^a,b^

| **Description** | **Accession** | **Accession length**  (aa) | ***AfOAH* from *Aspergillus fijiensis* CBS 313.89** | | | | | ***PlOAH* from *Penicillium lagena*** | | | | |
| --- | --- | --- | --- | --- | --- | --- | --- | --- | --- | --- | --- | --- |
|  |  |  | Max Score | Total Score | Query Cover (%) | E value | Identity  (%) | Max Score | Total Score | Query Cover (%) | E value | Identity  (%) |
| Hypothetical protein SS1G_08218 | [XP_001590478.1](https://www.ncbi.nlm.nih.gov/protein/XP_001590478.1?report=genbank&log$=prottop&blast_rank=1&RID=4EN0WPUR016) | 338 | 439 | 439 | 83 | 8.00E-156 | 70.28 | 387 | 387 | 93 | 1.00E-135 | 61.97 |
| Oxaloacetate acetylhydrolase | [AEY84228.1](https://www.ncbi.nlm.nih.gov/protein/AEY84228.1?report=genbank&log$=prottop&blast_rank=2&RID=4EN0WPUR016) | 249 | 382 | 382 | 72 | 2.00E-134 | 71.08 | 347 | 347 | 74 | 2.00E-121 | 69.79 |
| Oxaloacetate acetylhydrolase | [AEY84231.1](https://www.ncbi.nlm.nih.gov/protein/AEY84231.1?report=genbank&log$=prottop&blast_rank=3&RID=4EN0WPUR016) | 185 | 276 | 276 | 53 | 1.00E-93 | 69.02 | 256 | 256 | 57 | 2.00E-86 | 65.93 |
| Isocitrate lyase | [XP_001593548.1](https://www.ncbi.nlm.nih.gov/protein/XP_001593548.1?report=genbank&log$=prottop&blast_rank=4&RID=4EN0WPUR016) | 549 | 77.4 | 77.4 | 26 | 8.00E-16 | 46.74 | 76.6 | 76.6 | 34 | 1.00E-15 | 38.52 |
| Hypothetical protein SS1G_04900 | [XP_001593473.1](https://www.ncbi.nlm.nih.gov/protein/XP_001593473.1?report=genbank&log$=prottop&blast_rank=5&RID=4EN0WPUR016) | 550 | 69.3 | 69.3 | 24 | 4.00E-13 | 45.35 | 65.5 | 65.5 | 25 | 6.00E-12 | 40.96 |
| Hypothetical protein sscle_08g062640 | [APA11494.1](https://www.ncbi.nlm.nih.gov/protein/APA11494.1?report=genbank&log$=prottop&blast_rank=6&RID=4EN0WPUR016) | 598 | 69.3 | 69.3 | 24 | 5.00E-13 | 45.35 | 65.1 | 65.1 | 25 | 8.00E-12 | 40.96 |

^a^ The listed proteins were identified using the protein-protein BLAST (BLASTp) (Altschul et al. 1997, 2005) using *AfOAH* from *Aspergillus fijiensis* CBS 313.89 (GenBank Accession No. [XP_040799428.1](https://www.ncbi.nlm.nih.gov/protein/XP_040799428.1); 342 aa) or *PlOAH* from *Penicillium lagena* (GenBank Accession No. [XP_056833920.1](https://www.ncbi.nlm.nih.gov/protein/XP_056833920.1); 316 aa)as a query sequence against available data in GenBank, national center for biotechnology information website (NCBI, <http://www.ncbi.nlm.nih.gov/gene/>).

^b^ Listed proteins were used to generate the phylogenetic tree presented in Figure 1.

# References

Altschul, S. F., Madden, T. L., Schäffer, A. A., Zhang, J., Zhang, Z., Miller, W., et al. (1997). Gapped BLAST and PSI-BLAST: a new generation of protein database search programs. *Nucleic Acids Res.* 25, 3389–402.

Altschul, S. F., Wootton, J. C., Gertz, E. M., Agarwala, R., Morgulis, A., Schaffer, A. A., et al. (2005). Protein database searches using compositionally adjusted substitution matrices. *FEBS J.* 272, 5101–5109. doi: 10.1111/j.1742-4658.2005.04945.x.

Petzoldt, R., and Dickson, M. H. (1996). Straw test for resistance to white mold in beans . *Annu. Report-Bean Improv. Coop.* 39, 142–143.

Terán, H., Lema, M., Schwartz, H. F., Duncan, R., Gilbertson, R., and Singh, S. P. (2006). Modified Petzoldt and Dickson scale for white mold rating of common bean. *49th Annu. Rep. Bean Improv. Coop.* 49, 115–116.
